# Ultrasound‐Induced Nitric Oxide‐Propelled Nanomotor for Multimodal Theranostics of Cancer with Deep Penetration and Extended Lifetime

**DOI:** 10.1002/advs.202416709

**Published:** 2025-06-10

**Authors:** Xue Xu, Jinxu Cao, Yan Mu, Hao Zhang, Ya‐Lei Wang, Mengzhen Chen, Yuce Li, Qian Hua

**Affiliations:** ^1^ School of Medicine Wuhan University of Science and Technology Wuhan 430065 China; ^2^ School of Life Sciences School of Chinese Medicine Beijing University of Chinese medicine Beijing 100029 China; ^3^ College of Life Sciences and Health Wuhan University of Science and Technology Wuhan 430065 China

**Keywords:** nanomotor, nitric oxide therapy, tumor penetration, ultrasonic bioimaging

## Abstract

Cancer treatment is often ineffective due to poor bioimaging and resistance to standard therapies. This issue is exacerbated by multiple low‐penetrable bio‐barriers that limit the theranostic agents’ effectiveness in tumors. Here, a hollow nanomotor PM‐HMSN/Arg is fabricated by a sequential process involving: electrostatic adsorption of Mn^2+^, loading of l‐Arg, and coating of platelet membrane (PM), respectively. This nanomotor uses l‐Arg as an NO donor and ultrasound (US) as a trigger for NO release. After administration, it improves tumor penetration via a “tethering‐relaxing‐drilling” mechanism, overcoming bio‐barriers during delivery from blood vessels to tumor cells. NO regulates the metabolism of tumor vascular endothelial cells, facilitating relaxation, and enhances cytotoxicity by participating in reactive oxygen species metabolism. More importantly, the nanomotor's active motion enhances tissue penetration and retention in cancer, increasing therapeutic effects. In addition, continuous in situ NO generation extends US imaging signal lifetime. This innovative nanomotor shows promise for multimodal theranostics in low‐penetrable tumors.

## Graphical abstract

A hollow nanomotor (PM‐HMSN/Arg) propelled by ultrasound‐triggered nitric oxide (NO) was developed. The nanomotor employs a “tethering‐relaxing‐drilling” mechanism to overcome biological barriers, enabling multimodal theranostics of cancer with deep penetration and extended lifetime. It demonstrates significant anticancer efficacy through NO‐induced apoptosis and tumor microenvironment modulation, offering a promising strategy for treating low‐penetrable tumors.

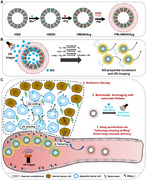

## 1 Introduction

Dynamic imaging‐guided precise theranostics of cancer holds considerable promise in clinical applications.^[^ ^1^, ^2^ ^]^ Nanomaterials can enhance tumor accumulation through active and passive targeting strategies, thereby improving imaging accuracy and therapeutic efficacy.^[^ ^3^, ^4^ ^]^ Several multifunctional nanomaterials, which combine imaging contrast and therapeutic capabilities, have demonstrated significant potential in tumor diagnosis and treatment.^[^ ^5^ ^]^ However, solid tumors often face the challenge of poor tissue penetration, as a result, nanoparticles tend to reach only the peripheral regions of the tumor, making it difficult to achieve accurate imaging and treatment of the entire tumor.^[^ ^6^ ^]^ This is caused by several biological barriers, including: the tumor vessel barrier with poor blood perfusion and compact endothelial lining, leading to a relatively low tumor vessel extravasation for most cargoes;^[^ ^7^, ^8^ ^]^ inordinately high interstitial fluid pressure (IFP) barrier derived from the abundant hyaluronan (HA) together with the large gel fluid phase in desmoplastic stroma, which forms the supreme obstacle to intratumor diffusion and convection, leaving the cargoes less mobile and trapped in tumor periphery.^[^ ^9^, ^10^, ^11^ ^]^

Most nanoparticles developed for cancer theranostics achieve tumor delivery through passive motion, such as free diffusion within the extracellular matrix, regardless of whether it is induced by passive or active targeting strategies.^[^ ^12^, ^13^ ^]^ As a result, the nanoparticles might be trapped in certain peripheral regions of the tumor that are less fluidic, such as the basement membrane.^[^ ^14^ ^]^ Synthetic nanoscale and microscale motors, capable of self‐propulsion, have garnered increasing attention due to their ability to actively transport cargo via non‐Brownian motion.^[^ ^15^, ^16^, ^17^, ^18^ ^]^ These nanomotors can undergo active motion driven by external forces or internal mechanisms, significantly enhancing their ability to traverse cell membranes and facilitate intracellular cargo delivery.^[^ ^19^, ^20^ ^]^ In addition, nanomotors can improve their movement within the tumor microenvironment (TME), increasing the likelihood of recognizing and binding to tumor cells and tissue components, thus enhancing tumor selectivity.^[^ ^21^ ^]^ More importantly, the substantial thrust generated by nanomotors enables them to overcome various barriers within tumor tissues, improving tissue penetration capabilities.^[^ ^22^ ^]^ These properties make nanomotors an excellent nanoplatform for tumor diagnosis and treatment.

Ultrasonic (US) imaging is a commonly used imaging technique available for detection or intraoperative assessment of cancer extension. However, US imaging mediated by some nanomaterials usually provides unsatisfactory benefits because the nanobubbles extravasated into tumors are unstable, which collapse and leak gas rapidly, leading to premature US diagnosis termination.^[^ ^23^, ^24^ ^]^ Even though many studies have focused on overcoming gas depletion problem by increasing gas loadings,^[^ ^25^, ^26^ ^]^ they fail to overcome the inherent capacity limitation of the nanoscale core to carry sufficient gases. Thus, it is crucial to improve the duration of gas generation to extend the lifetime of US signals and thereby improve the imaging capability.

**Scheme 1:**
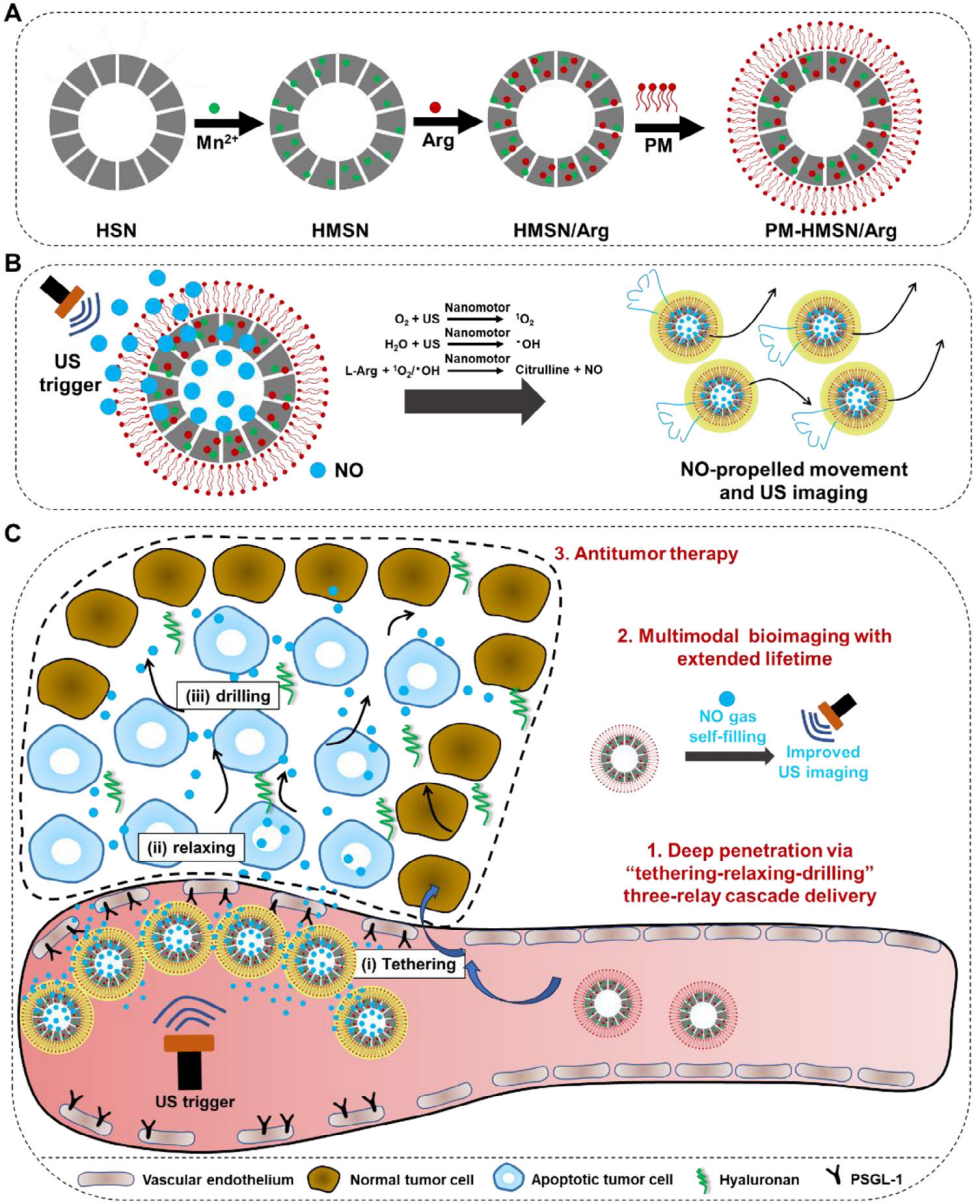
Design features and proposed action mechanism of PM‐HMSN/Arg in vivo. A) Schematic of PM‐HMSN/Arg which is constructed through typical layer‐by‐layer coatings method. B) The US‐powered and NO‐propelled motion mechanism of PM‐HMSN/Arg serving as nanomotor. Briefly, through energy transformation from US mechanical energy to chemical energy of reactive oxygen species (ROS) occurred on manganese silicate walls, the entrapped l‐Arg can be oxidized into NO for both self‐filling in hollow core and extravasating outside to propel the nanomotors active movement. C) PM‐HMSN/Arg combined with US trigger overcoming multiple bio‐barriers in a way of “tethering‐relaxing‐drilling” three‐relay cascade delivery, which induces multimodal theranostics of cancer with deep penetration and extended lifetime.

In this work, we developed a nanomotor for multimodal theranostics of cancer with improved penetration in tumor tissue and extended imaging lifetime. This nanomotor consists of a biomimetic platelet membrane (PM) surface, which allows rapid and specific adherence to tumor vessels, forming stable tumor vessel retention,^[^ ^27^, ^28^, ^29^ ^]^ and a robust nitric oxide (NO) nanogenerator that produces a large amount of NO to propel active movement. As a star molecule in tumor gas therapy, NO participates in various metabolic activities in tumor tissues, including but not limited to the relaxation of tumor vascular endothelial cells, intracellular reactive oxygen species (ROS) metabolism, DNA damage, nitrosylation of heme iron core, and participation in immune responses. As the motion energy source of the nanomotor, NO was generated during US treatment by the following mechanism: with the mechanical energy of US, ROS, including singlet oxygen (^1^O_2_) and hydroxyl free radicals (•OH), were generated through the electron‐hole separation induced by hollow manganese silicate nanoparticle (HMSN); subsequently, l‐Arginine (l‐Arg) was oxidized by the generated ROS, yielding NO and citrulline.^[^ ^30^ ^]^ These NO bubbles aggregate on the surface of the nanomotor through microscopic interaction, and then separate from the nanomotor when the nanobubbles reach a threshold, providing energy for the movement of the nanomotors.^[^ ^31^, ^32^ ^]^ The above structure of the nanomotor significantly improved tumor penetration ability by a distinct “tethering‐relaxing‐drilling” three‐relay cascade delivery. That is, the nanomotor first rapidly and specifically recognized and anchored to tumor vessels by the affinity with PM. Then, the US‐induced NO molecules diffused into the tumor tissue, resulting in the relaxation of endothelial cells. Subsequently, these nanomotors drill into deep tumor tissues through NO‐driven active movement. Moreover, as the “exhaust” of the nanomotor, NO was continuously generated in situ during US treatment, largely extending the lifetime and enhancing the intensity of US imaging signals due to the gas self‐filling capability. Therefore, this hollow nanomotor could penetrate tumors deeply and intelligently take advantage of the NO self‐filling capability for multimodal theranostics in low‐penetrable tumors (**Scheme** **1**).

## 2 Results and Discussion

### 2.1 Preparation and Characterization of PM‐HMSN/Arg

**Figure 1:**
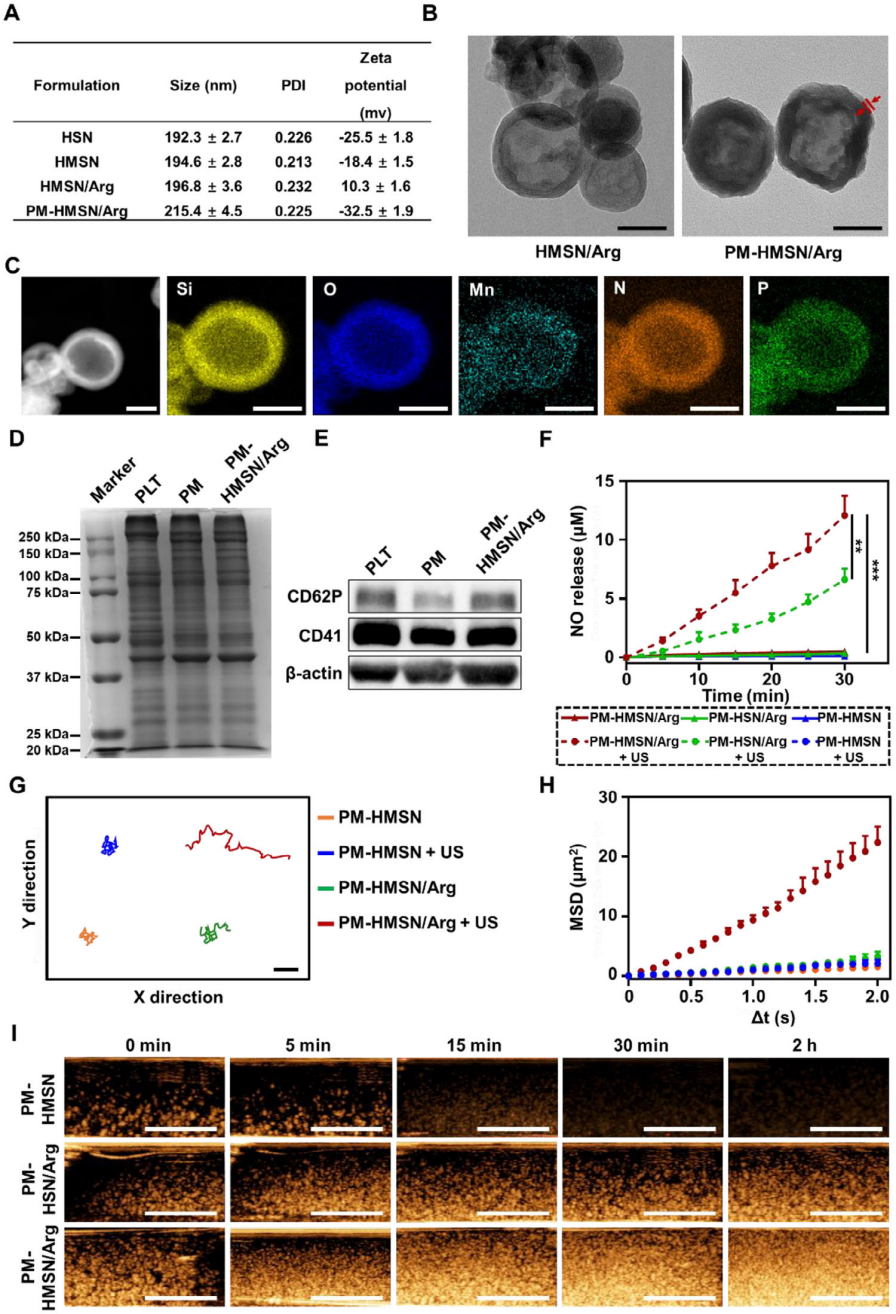
Characterization of PM‐HMSN/Arg. A) Particle size, polydispersity index (PDI), and zeta potential of HSN, HMSN, HMSN/Arg, and PM‐HMSN/Arg, respectively (*n* = 3). B) TEM images of HMSN/Arg and PM‐HMSN/Arg. Scale bar: 100 nm. C) Element mapping for silica, oxygen, manganese, nitrogen, and phosphorus of PM‐HMSN/Arg. Scale bar: 100 nm. D) SDS‐PAGE analysis of the broad protein profile and E) western blot analysis of specific CD41 and CD62P protein expression in purified platelets, platelet membranes, and PM‐HMSN/Arg. F) In vitro NO generation and release in PBS after incubation with different formulations. G) Motion behavior assay by optical tracking trajectories and H) mean‐squared displacement (MSD) of PM‐HMSN or PM‐HMSN/Arg in PBS solution with/without US treatment (*n* = 20). Scale bar: 5 µm. I) In vitro ultrasound images of 1 mg mL^−1^ of different nanosphere suspensions in physiological saline solution recorded at different imaging times. Scale bar: 1 cm. Data are presented as mean ± SD. **p < 0.01, ***p < 0.001 among the marked groups using nonparametric two‐tailed analysis of variance.

Hollow silica nanosphere (HSN) was synthesized through a modified aerosol‐induced self‐assembly method, and PM‐HMSN/Arg was constructed through a sequential process involving: electrostatic adsorption of Mn^2+^, loading of l‐Arg, and coating of PM, respectively. The main pore diameter of HSN was around 4.3 nm (Figure S1, Supporting Information). The loading of l‐Arg with a 24.2% content led to the slight positive charge of HMSN/Arg, and the PM coating reversed its surface charge into negative (−32.5 mV) and resulted in ≈20 nm diameter increase to 215.4 nm (**Figures** **1**A and S2, Supporting Information). The content of Mn and PM were 4.8% and 24.2%, respectively, which were determined by ICP‐MS and TGA (Figure S3, Supporting Information). The transmission electron microscope (TEM) images verified the hollow structure of PM‐HMSN/Arg and the PM coatings (≈8 nm) on the surface (Figure 1B). The mapping of Si, O, Mn, N, and P element (Figure 1C) also demonstrated their successful incorporation. As shown in the Fourier‐transform infrared (FTIR) spectra (Figure S4, Supporting Information), the strong bands at 965–1275 cm^−1^ of HSN affiliated with the Si─O asymmetric stretching, while the bands at 805 and 464 cm^−1^ are attributed to the Si─O symmetric stretching and the Si─O─Si bending modes, respectively. Two characteristic peaks at 1679 and 1329 cm^−1^ are observed in HMSN/Arg and ascribed to the bands of l‐Arg. PM‐HMSN/Arg displayed the characteristic absorption peaks of l‐Arg, along with the peaks at ≈2925 cm^−1^ that belong to the ν_as_ of alkyl C─H derived from the lipids in PM, indicating the successful layer‐by‐layer coatings. The SDS‐PAGE analysis displayed that the protein profile of PM‐HMSN/Arg was similar to that of PM (Figure 1D), suggesting that the proteins were almost completely retained during the preparation of PM‐HMSN/Arg. Importantly, western blot analysis validated the presence of specific PM markers (CD41 at 113 kDa and CD62P at 140 kDa, respectively) (Figure 1E), which act as the crucial receptor for tumor vascular endothelium ligand, such as P‐selectin glycoprotein ligand‐1 (PSGL‐1). In addition, the particle size remained constant with time and US irradiation, revealing good physical stability and mechanical tolerance, which is favorable for the following study (Figures S5, S6, Supporting Information).

It is worth noting that PM‐HMSN serves as a ROS generation platform, which could be excited by US irradiation for electron–hole separation, and then interact with O_2_ and H_2_O to produce massive ^1^O_2_ and •OH, respectively, via energy transformation (Figures S7, S8, Supporting Information).^[^ ^34^ ^]^ Subsequently, the resultant ROS could oxidize l‐Arg into citrulline and produce NO (Scheme 1B), which was validated by the Griess assay (Figure 1F). Regardless of US treatment, PM‐HMSN had negligible NO release, indicating the essential role of l‐Arg as an NO donor. Compared to the low NO level in the PM‐HMSN/Arg group (0.49 × 10^−6^ m), US treatment significantly increased the NO level (12.07 × 10^−6^ m), supporting US‐induced NO generation. The generation of NO by the nanomotor under US treatment nearly follows a zeroth order reaction and the NO generation rate was calculated as 0.40 × 10^−6^ m min^−1^. Notably, the difference between PM‐HMSN/Arg and PM‐HSN/Arg showed the enhanced NO yielding by the Mn element because the low‐valence Mn was partially oxidized to high valence, promoting the electron–hole separation as well as more ROS for l‐Arg oxidation.^[^ ^35^, ^36^ ^]^ To confirm this, we performed XPS analysis of the nanomotor after ultrasonic irradiation. As shown in Figure S9 (Supporting Information), the peak between 640 and 647 eV belongs to the 2p_3/2_ of Mn element and the peak between 650 and 657 eV belongs to the 2p_1/2_ of Mn element. The peaks could be divided into two pairs of peaks, which could be assigned to Mn^IV^ (654.2 and 642.6 eV) and Mn^II^ (652.8 and 641.2 eV). These results indicated the existence of both Mn^2+^ and Mn^4+^ after ultrasonic treatment.

To evaluate the dynamic motion of PM‐HMSN/Arg propelled by the generated NO, we acquired the optical tracking data and calculated the mean‐squared displacement (MSD) accordingly (Figure 1G,H). PM‐HMSN diffuses randomly by Brownian motion without any directionality regardless of the US irradiation (Video S1,S2, Supporting Information). In contrast, PM‐HMSN/Arg showed similar Brownian motion without US treatment but moved rapidly under US irradiation (Video S3,S4, Supporting Information), with the corresponding MSD increasing to velocities of 0.35 and 2.60 µm s^−1^, respectively, validating the active movement capability of PM‐HMSN/Arg propelled by NO. Given its symmetrical structure, we speculated that the US‐inspired NO‐driven motion can be realized through microscopic interactions on the surface of this nanomotor. Briefly, small NO bubbles can be created by the nanomotors, and when two gas bubbles attract each other, they coalesce into one. This process is constantly reproduced, so that a large bubble can be formed by the small ones. When the large bubble reaches a maximum radius, it detaches from the nanoparticle and provides energy for the movement of the nanomotor.^[^ ^32^, ^37^ ^]^

To validate the capacity of this nanomotor as a US imaging reagent with extended lifetime and enhanced signal, we evaluated the time‐dependent US imaging in vitro (Figure 1I). PM‐HMSN only performed transient US imaging within 15 min, which benefited from the initially retained air inside the hollow cavity, but diminished gradually due to the unavoidable gas depletion. In contrast, the l‐Arg‐containing formulations displayed prolonged US signals for at least 2 h. In particular, PM‐HMSN/Arg maintained a stronger reflection signal compared to PM‐HSN/Arg, which is related to the stronger NO gas production due to the higher ROS generation. This self‐filling mechanism in the hollow core of the nanomotor allows for gas supplementation and addresses the issue of gas depletion and premature bioimaging termination, leading to extended and improved US imaging capability.

Taken together, the nanomotor PM‐HMSN/Arg was successfully constructed and is capable of generating NO to propel active movement while simultaneously exerting function‐enhanced US imaging under external US treatment.

### 2.2 Delivery and Antitumor Effect of PM‐HMSN/Arg In Vitro

**Figure 2:**
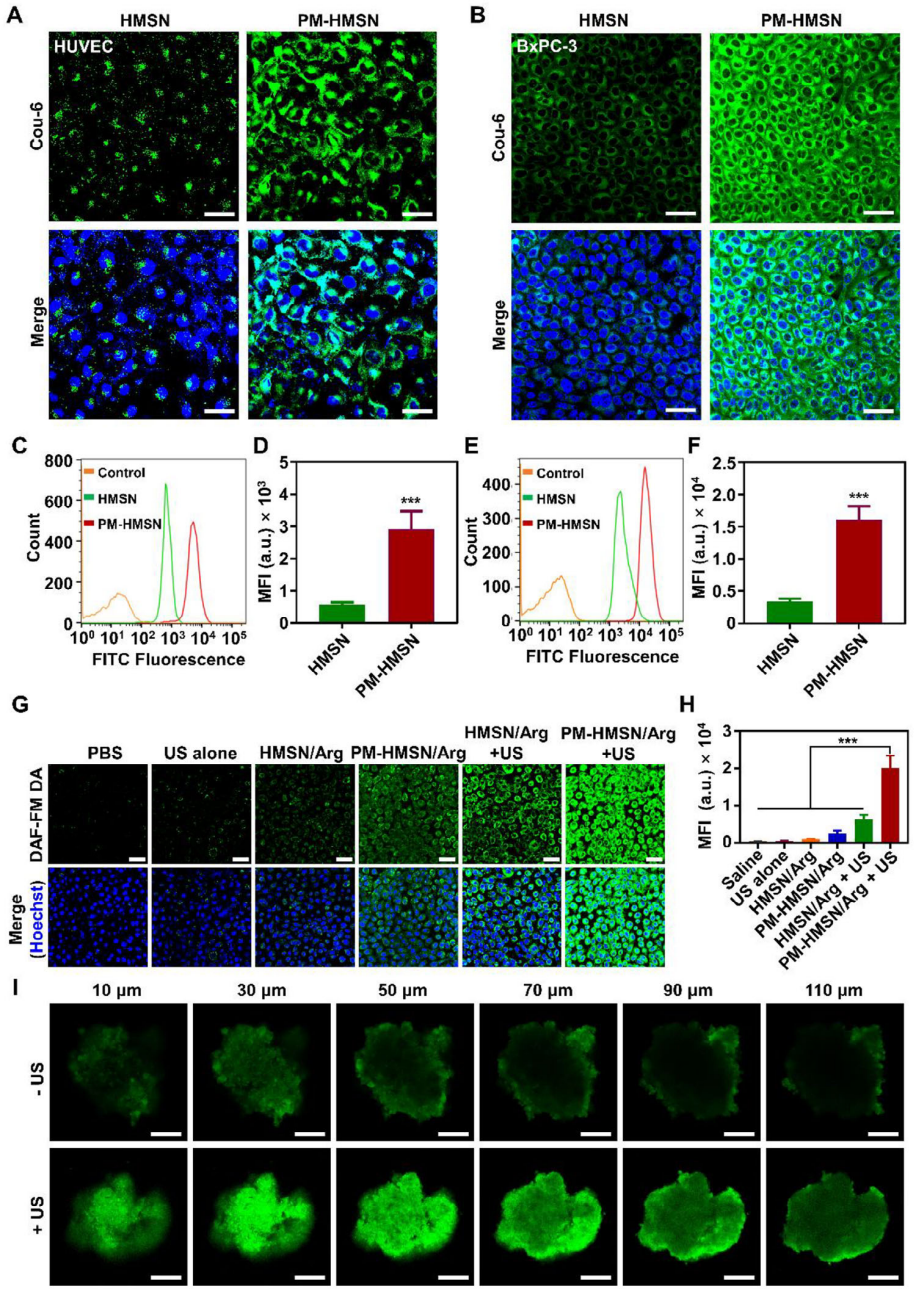
In vitro delivery of PM‐HMSN/Arg. A,B) CLSM image of HUVEC cells and BxPC‐3 cells incubated with HMSN and PM‐HMSN. C) Flow cytometry analysis of HUVEC cells incubated with HMSN and PM‐HMSN, respectively, and D) mean fluorescence intensity (MFI) calculated from panel (C) (*n* = 3). E) Flow cytometry analysis of BxPC‐3 cells incubated with different nanomotors, and F) MFI calculated from panel (E) (*n* = 3). G) Representative fluorescence images of intracellular NO distribution in BxPC‐3 tumor cells and H) the corresponding semiquantitative MFI of NO probe DAF‐FM DA (*n* = 6). I) The Cou‐6 labeled nanomotor PM‐HMSN/Arg penetration into BxPC‐3 & NIH3T3 (B&N) hybrid 3D tumor spheroids with/without US treatment. Scale bar: 200 µm.

In a sense, tumors are regarded as a kind of wound in the human body that is difficult to heal. As a result, platelets have the affinity to tumor vessels and tumor cells. We explored the targeting binding ability of PM‐coated nanomotors with tumor vessel endothelial cells (activated human umbilical vein endothelial cells [HUVEC] cells) and pancreatic tumor cells (BxPC‐3 cells), respectively (**Figure** **2**A–F). The green fluorescent signal intensity of HUVEC cells treated with coumarin‐6 (Cou‐6) labeled PM‐HMSN was higher than that of HMSN (Figure 2A), and about a 5.1‐fold increase was confirmed by flow cytometry (Figure 2C,D). Similarly, PM‐HMSN also displayed a 4.8‐fold higher binding to BxPC‐3 tumor cells compared to the HMSN group (Figure 2B,E,F). Clearly, the PM coating improved the targeting affinity of HMSN for tumor vessel endothelial cells and tumor cells through its intrinsic biomimetic advantage, which would facilitate tethering to the tumor vessel after intravenous administration.

Subsequently, the intracellular NO release was determined using the DAF‐FM DA probe (Figure 2G,H). Neither US nor the nanomotor alone elevated intracellular NO significantly due to the lack of the energy source or an NO donor to yield NO. In contrast, PM‐HMSN/Arg + US displayed the most remarkable intracellular NO production, attributed to the favorable adhesion and endocytosis mediated by the biomimetic PM coating as well as the US‐induced ROS. To evaluate the tumor penetration of the nanomotor, we established stroma‐rich BxPC‐3 & NIH3T3 (B&N) hybrid 3D tumor spheroids to simulate the intratumor permeability barrier, and cultured them with PM‐HMSN/Arg for in vitro penetration evaluation (Figure 2I). Only a few green fluorescence signals were distributed on the periphery of the tumor spheroid without US irradiation, while remarkably deeper and wider diffusion of PM‐HMSN/Arg into the central region was obtained under US irradiation. This result was attributed to the synergistic effect of NO‐induced relaxation and the NO‐propelled active motion of the nanomotor described in Figure 1H, which countered the diffusion resistance within the tumor spheroids.

**Figure 3:**
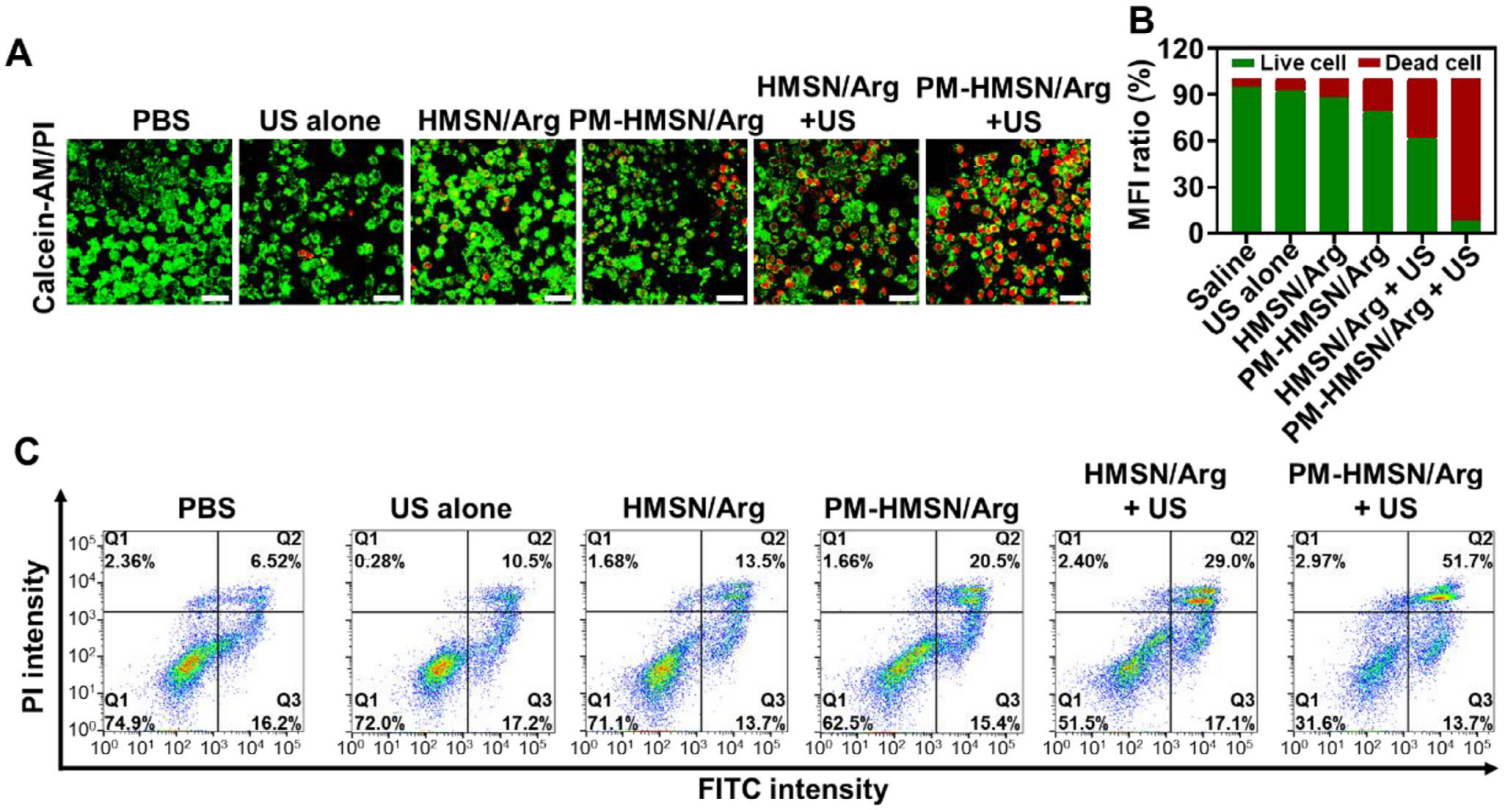
In vitro antitumor effects of PM‐HMSN/Arg. A) Confocal images of BxPC‐3 tumor cells stained with calcein‐AM (green) and propidium iodide/PI (red) after different treatment, and B) corresponding semiquantitative analysis (*n* = 3). Scale bar: 50 µm. C) Flow cytometry data of BxPC‐3 cells stained by annexin V‐FITC and PI after different treatment. Data are presented as mean ± SD. ***p < 0.001 among the marked groups using nonparametric two‐tailed analysis of variance.

Furthermore, we evaluated the antitumor effect in vitro by live‐dead cell staining (Figure 3A,B) and flow cytometry analysis (**Figure** **3**C). The proportions of dead cells ranked as follows: phosphate buffer saline (PBS) (5.12%) < US alone (7.91%) < HMSN/Arg (12.3%) < PM‐HMSN/Arg (21.07%) < HMSN/Arg + US (38.18%) < PM‐HMSN/Arg + US (91.47%). US treatment did not cause significant cell injuries compared to PBS treatment due to its mild irradiation intensity. Consistent with the intracellular NO content, PM‐HMSN/Arg with US trigger caused the highest extent of cell death, validating its strong killing effect on tumor cells. Furthermore, the mechanism was investigated by flow cytometry analysis (Figure 3C) according to previous reports indicating that NO mediates remarkable tumor cells apoptosis via multiple pathways (e.g., upregulating P53 and caspase‐3 pathways).^[^ ^38^, ^39^, ^40^ ^]^ Compared to the PBS group, nanomotors with NO generation capability induced significantly more apoptosis. Among them, the proportion of late apoptotic BxPC‐3 cells after treatment with PM‐HMSN/Arg + US was 51.7%, much higher than in other groups, implying concentration‐dependent tumor cells apoptosis induced by NO. The above cytotoxicity experiments confirmed the therapeutic potential of PM‐HMSN/Arg by NO‐induced tumor cell apoptosis.

Hence, PM‐HMSN/Arg can effectively tether to the HUVEC and BxPC‐3 cells through PM biomimetic modification, generate a large amount of NO to relax the tissue, and propel to facilitate deeper penetration, which is the so‐called “tethering‐relaxing‐drilling” three‐relay cascade delivery. Finally, the nanomotor achieved potent killing effects on tumor cells via NO‐mediated cellular apoptosis.

### 2.3 Three‐Relay Cascade Delivery Mediated by PM‐HMSN/Arg In Vivo

Many cancers, including pancreatic cancer, are characterized by poor blood perfusion, low vessel permeability, dense extracellular matrix, and high IFPs, compromising the delivery of most cargoes into tumors. We investigated the tumor delivery efficacy of PM‐HMSN/Arg in a low penetrable subcutaneous BxPC‐3 xenograft mouse model.

**Figure 4:**
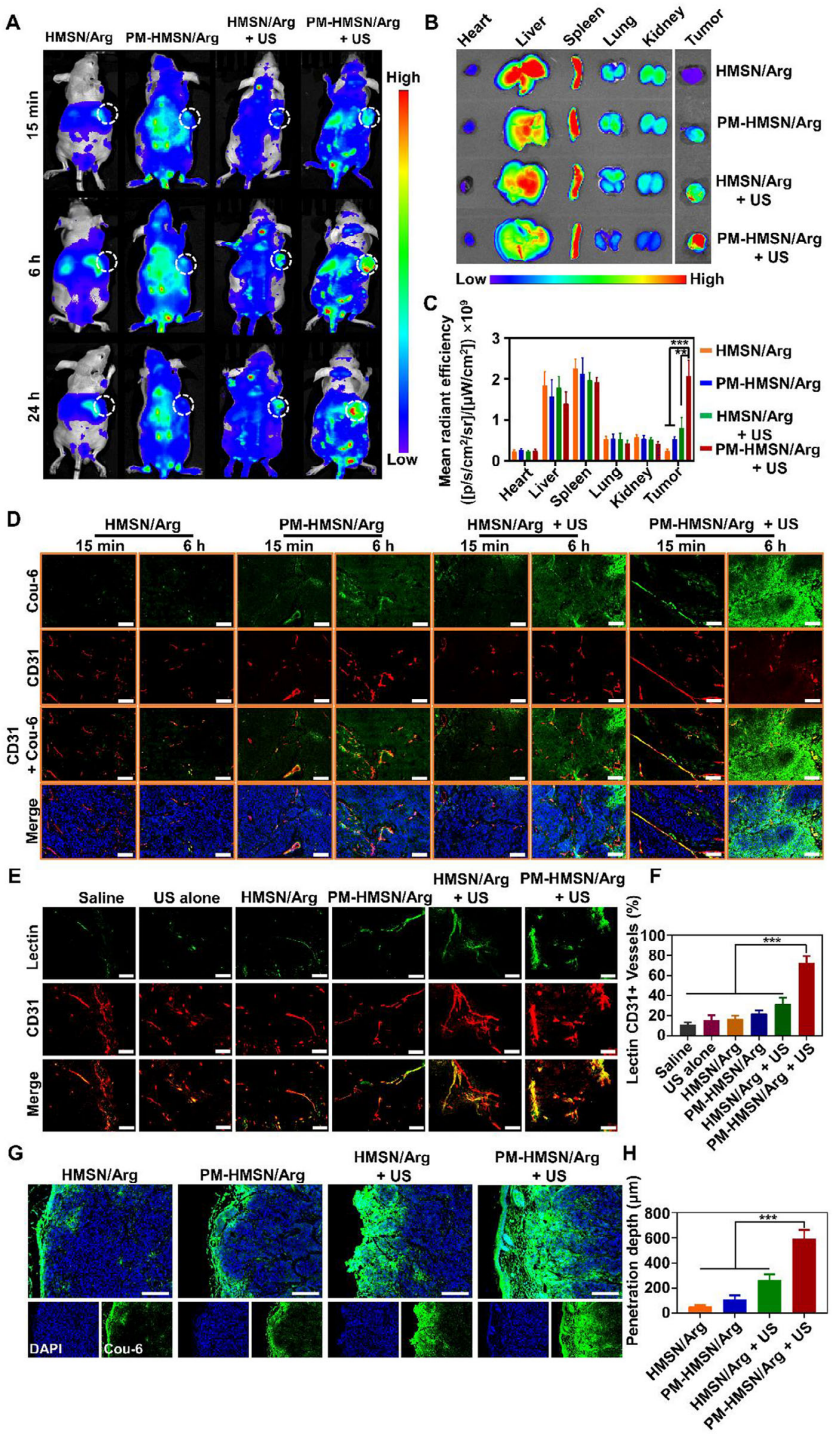
Deep tumor penetration of PM‐HMSN/Arg by three‐relay cascade delivery. A) Biodistribution of DiR‐labeled nanomotor at 15 min, 6 h, and 24 h after intravenous injection in subcutaneous BxPC‐3 tumor‐bearing mice. US were applied to the tumor tissues using a portable focused ultrasound therapeutic apparatus DJO‐2776 sonicator, with the parameters of 1.0 MHz‐1.0 W‐20% for 15 s with a 30 s interval between cycles, and 20 cycles of US irradiation. B) Ex vivo images of each organ collected from mice at 24 h post‐injection. C) Semiquantitative fluorescence intensity of each organ (heart, liver, spleen, lung, kidney, and tumor) at 24 h post injection (*n* = 3). D) Intratumor distribution of Cou‐6‐labeled nanomotor monitored via immunofluorescent staining of tumor sections. Green: Cou‐6. Red: CD31. Blue: DAPI. Scale bar: 100 µm. E) FITC‐labeled lectin perfusion along with CD31 marking the tumor vessels. Scale bar: 200 µm. F) The proportion of lectin CD31+ vessels (*n* = 5). G) Distribution of nanomotors within the tumor at 6 h post‐injection and H) the corresponding penetration depth of nanomotors toward the tumor core calculated using ImageJ software (*n* = 5). Green: Cou‐6; Blue: DAPI. Scale bar: 200 µm. Data were represented as mean ± SD. **p < 0.01, ***p < 0.001, among the marked groups using nonparametric two‐tailed analysis of variance.

We monitored the overall in vivo biodistribution of the nanomotor using the IVIS imaging system with the near‐infrared fluorescent dye DiR‐encapsulated PM‐HMSN/Arg (**Figure** **4**A–C). There was no significant difference in the fluorescence intensity of DiR in major organs among each group. However, PM‐HMSN/Arg + US treated mice showed markedly superior in vivo and ex vivo tumor distribution, with tumor accumulation at 24 h being about 8.5‐, 3.8‐, and 2.6‐fold higher than that of HMSN/Arg, PM‐HMSN/Arg, and HMSN/Arg + US, respectively. Both the PM coating and US activation contributed to the tumor‐specific accumulation. More importantly, given that (1) the targeted enrichment of PM‐HMSN/Arg at tumors, (2) the fact that US irradiation can be narrowly focused on the tumor site to induce NO release, and (3) the short diffusion distance of NO due to its rapid metabolic deactivation and short half‐life in vivo, we assume that PM‐HMSN/Arg mediated NO therapy could be predominately confined within tumors. This confinement guarantees a relatively specific oncotherapy with diminished NO generation and reduced exposure damages to adjacent healthy tissues or normal organs (e.g., liver and spleen).

We assume that the above improvement can be attributed to the aforementioned distinct three‐relay cascade delivery as follows: (1) Tethering: biomimetic PM‐HMSN/Arg could extend its circulation time and rapidly bind with tumor vessels for primary enrichment from the blood to tumor vessels; (2) Relaxing: the focused US treatment led to localized NO generation at the tumor site to relax the tumor matrix, allowing for more perfusion and extravasation of nanomotors, which assist their permeatment from tumor vessels to the extracellular matrix; (3) Drilling: the US‐powered and NO‐propelled nanomotors could actively drill into the tumors against the high IFPs and across the stroma, allowing for more effective tumor cell endocytosis, which improved the delivery from interstitial matrix to tumor cells (Scheme 1).

To demonstrate the tethering of PM‐coated nanomotors and intratumor delivery route, we performed the immunofluorescent staining of tumor sections at different time points post‐injection of Cou‐6‐encapsulated PM‐HMSN/Arg. Due to the strong affinity of PM to the tumor vessels, a large amount of the PM‐coated nanomotors (PM‐HMSN/Arg) were observed colocalized with tumor vessels (CD31‐positive) only 15 min after intravenous injection (Figure 4D). The rapid tumor accumulation of PM‐HMSN/Arg within 15 min post‐injection can be attributed to the PM‐mediated active targeting through the affinity between CD41/CD62P on PM and endothelial markers on tumor‐associated vasculatures such as P‐selectin glycoprotein ligand‐1 (PSGL‐1). This mechanism avoids the limitations of conventional passive EPR effect, allowing for the rapid “tethering” of the nanomotors to the tumor vessels.

Thereafter, US treatment was conducted for NO generation to assist intratumor delivery. At 6 h, more extravasation and deeper distribution of green Cou‐6 fluorescence within tumors was observed in the PM‐HMSN/Arg group (Figure 4D), which is consistent with the trends in Figure 2I, indicating that NO‐propelled nanomotors facilitated the intratumor permeability. To affirm the role of NO‐induced relaxing (tumor vasodilation) in this cascade delivery, we conducted the FITC‐labeled lectin perfusion along with CD31 marking the tumor vessels (Figure 4E,F). Only a minimal proportion of FITC‐labeled lectin/CD31 vessels (10.6%) was observed in the saline group, indicating poor blood perfusion, which was in accordance with the sparse and diminished functional vasculature characteristics of hypo‐perfused pancreatic cancer. US or nanomotors alone did not induce significantly improvement in the functional tumor vessels. When combined with US, tumor perfusion increased, especially the PM‐HMSN/Arg + US group possessed the highest functional tumor vessel ratio (72.8%), confirming the vasodilation effect of PM‐HMSN/Arg by NO release. Thes result affirmed the “relaxing” on the tumor vessels mediated by the nanomotors.

Furthermore, we evaluated the penetration depth of these nanomotors in vivo (Figure 4G,H). Collecting the tumor tissues at 6 h post‐injection, we observed that green fluorescence signals (Cou‐6 labeled nanomotors) were only distributed around the periphery of the tumor in US‐absent groups, indicating limited penetration without active movement propelled by NO. In contrast, significantly intense green fluorescence was observed in deeper regions in the US‐treated groups. Particularly, PM‐HMSN/Arg + US caused the deepest penetration with a depth around 591.4 µm, which was about 11.1‐, 5.3‐, and 2.3‐fold higher than HMSN/Arg, PM‐HMSN/Arg, and HMSN/Arg + US, respectively. These results revealed the advantage of both biomimetic PM coating and NO propelling for tumor deep penetration via three‐relay cascade delivery.

Collectively, PM‐HMSN/Arg can deeply penetrate into tumors by successively overcoming multiple bio‐barriers in a manner of three‐relay cascade delivery, paving the way for subsequent diagnosis and therapy.

### 2.4 In Vivo NO Release and Lifetime‐Extended US/T1‐MRI Dual‐Modality Imaging

**Figure 5:**
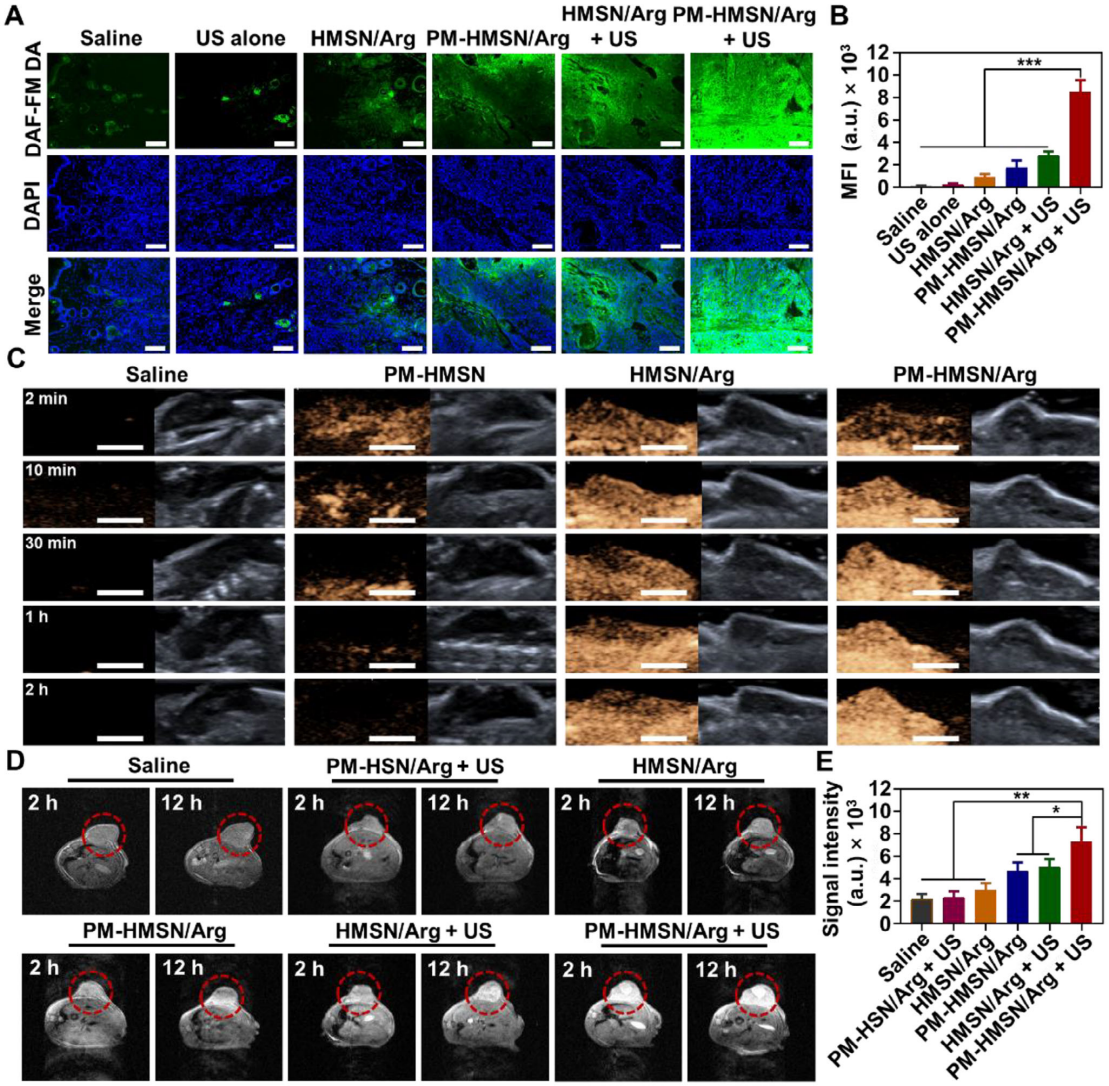
US triggered NO release and US/T1‐MRI dual‐modality imaging by PM‐HMSN/Arg in vivo. A) Intratumor NO release labeled by DAF‐FM DA. Green: DAF‐FM DA. Blue: DAPI. Scale bar: 50 µm. B) Semiquantitative fluorescence intensity of DAF‐FM DA (*n* = 6). C) In vivo US imaging to monitor the tumor region. Scale bar: 5 mm. D) In vivo T1‐MRI and E) the corresponding signal intensity within tumors at 12 h after intravenous injection of different formulation (*n* = 3). Data were represented as mean ± SD. *p < 0.05, **p < 0.01, ***p < 0.001, among the marked groups using nonparametric two‐tailed analysis of variance.

The BxPC‐3‐bearing nude mice were intratumorally injected with DAF‐FM DA after different formulations treatment to evaluate the intratumor NO release level. As shown in **Figure** **5**A,B, these nanomotors without US treatment negligibly improved the intratumor NO level, consistent with their low intracellular NO release in Figure 2H. PM‐HMSN/Arg + US showed the highest intratumor DAF‐FM DA fluorescence intensity with a wider and more homogeneous distribution, benefiting from its deep tumor penetration via three‐relay cascade delivery.

Subsequently, we evaluated the US imaging effect on tumor tissues by the nanomotor (Figure 5C). US imaging is usually unsatisfactory because the signals are unstable, leading to premature US diagnosis termination. Although PM‐HMSN exhibited a transient US imaging within initial 10 min, which is much better than the saline group that lacked obvious US reflection signal, this signal rapidly diminished along with the depletion of air within the inner core under complex pressures such as spontaneous gas diffusion and dissolution in the aqueous microenvironment. In comparison, PM‐HMSN/Arg displayed much more stable US imaging signals with the diagnosis lifetime extended to at least 2 h, and maintained high signal intensity throughout the whole test, consistent with their in vitro US imaging capability in Figure 1I. Its excellent US imaging performance can be attributed to two key points: (1) the targeted accumulation and deep tumor penetration of theranostic agents with superior biodistribution for ready US imaging; (2) the US‐responsive NO could be self‐filled in the hollow core of nanomotors as a gas supplement to maintain sustainable acoustic reflection. Furthermore, based on the NO‐mediated US imaging, the delivery process and NO yielding degree might be observed simultaneously, contributing to potential real‐time NO therapy surveillance.

Nevertheless, US imaging can generally obtain rough tumor size, shape, and location, but it is still difficult to precisely determine the edge between tumor and normal tissues. As reported, Mn element can mediate a T1‐weighted MRI,^[^ ^41^ ^]^ assisting in obtaining more abundant tumor information. As shown in Figure S10 (Supporting Information), PM‐HMSN/Arg possesses a T1‐weighted MRI capability with a Mn concentration‐dependent and pH‐sensitive behavior. The in vivo T1‐weighted MRI results indicated that the Mn‐doped formulations had obvious MRI signals, and the PM‐HMSN/Arg + US group showed the brightest T1‐MRI signal enhancement depending on its abundant accumulation within tumors (Figure 4D,E). In contrast, saline or Mn‐absent PM‐HSN/Arg group with weak T1‐MRI imaging.

Therefore, US‐triggered nanomotor PM‐HMSN/Arg could release massive NO into tumors, along with the guidance of improved US/T1‐MRI dual‐modality imaging.

### 2.5 In Vivo Anticancer Effect of PM‐HMSN/Arg

Considering the superior intratumor distribution of NO that has inherent potential to induce tumor cell apoptosis, we anticipated a promising antitumor effect by PM‐HMSN/Arg. Here, a subcutaneous BxPC‐3 mouse pancreatic tumor model is adopted, considering the strong dependence of pancreatic cancer patients on US diagnosis in the clinic (e.g., sonographic visualization of tumor tissues, tissue acquisition, and staging of pancreatic cancer). Compared to the orthotopic pancreatic tumor, the BxPC‐3 tumor model possesses typical bio‐barriers, including both less leaky tumor vessels and dense desmoplastic stroma, enabling rational evaluation of the benefits of three‐relay cascade delivery and the antitumor outcomes.

**Figure 6:**
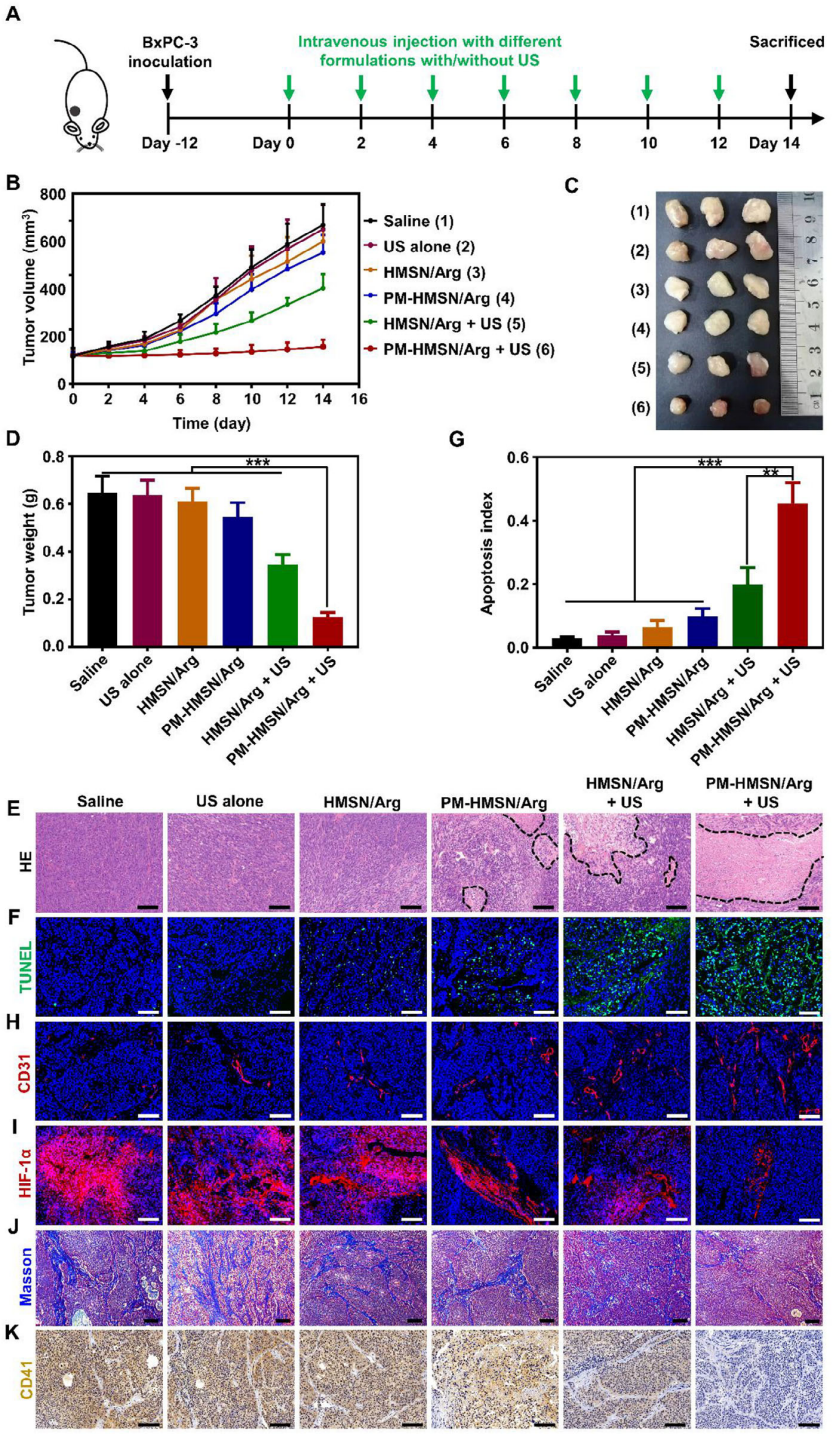
In vivo antitumor effect of US activated PM‐HMSN/Arg in a BxPC‐3 pancreatic tumor model. A) Scheme of antitumor therapy. B) Tumor growth curves during treatment (*n* = 6). C) Representative tumor xenograft images. D) Average tumor weights at the end of each treatment (*n* = 6). E) H&E staining and F) TUNEL staining of tumor slices in different groups. Blue: DAPI. Green: TUNEL. G) Quantitative analysis of cell apoptosis rates from the TUNEL assay (*n* = 3). H) Representative fluorescence images of tumor neovascularization after this therapy. Blue: DAPI for staining cell nucleus. Red: CD31 for staining tumor neo‐vasculature. Scale bar: 100 µm. I) Immune‐staining of HIF‐1α. Scale bar: 100 µm. J) Masson's trichrome staining for type I collagen (blue) examination. Scale bar: 100 µm. K) Immunohistochemical staining of CD41 (brown). Scale bar: 100 µm. Data are presented as mean ± SD. **p < 0.01, ***p < 0.001, among the marked groups using nonparametric two‐tailed analysis of variance.

The treatment was conducted as shown in **Figure** **6**A. Since treatment started, different formulations were injected, and the tumor tissues were treated with US radiation every two days. The tumor growth curve of the US alone group almost overlapped that of saline group, indicating its negligible tumor suppression activity (Figure 6B). Single HMSN/Arg and PM‐HMSN/Arg treatment only slightly inhibited tumor growth due to the lack of US‐induced NO. However, when combined with US, the antitumor activity of the nanomotors was significantly amplified, and the PM‐HMSN/Arg + US group showed the strongest tumor suppressive effect. The results were further confirmed by photographs and the inhibition rate of tumor (IRT) calculated based on the average tumor weights (Figure 6C,D). Notably, the IRT of the PM‐HMSN/Arg + US treated mice (80.44%) was much higher than that of the mice treated with US alone (0.61%), HMSN/Arg (3.42%), PM‐HMSN/Arg (13.62%), and HMSN/Arg + US (46.05%), respectively.

Next, to uncover the mechanism enabling the antitumor activity of PM‐HMSN/Arg, the tumor tissues were collected for hematoxylin and eosin (H&E) staining and TUNEL assay (Figure 6E,F). Massive nuclei damage and cytosol degradation were observed in PM‐HMSN/Arg + US therapy, indicating marked apoptosis of tumor cells. According to the TUNEL assay in Figure 6G, PM‐HMSN/Arg + US induced the highest degree of tumor cell apoptosis, with a 15.2‐, 11.2‐, 7.0‐, 4.6‐, and 2.3‐fold higher apoptosis index (0.454) than that of the saline (0.030), US alone (0.041), HMSN/Arg (0.065), PM‐HMSN/Arg (0.099), and HMSN/Arg + US groups (0.199), respectively. Collectively, PM‐HMSN/Arg served as a US‐powered nanomotor can effectively induce tumor apoptosis via controllable NO release.

Besides its anticancer activity, NO can also remodel the TME to further improve its oncotherapy potential. First, a high concentration of NO could not only kill tumor cells but also promote newborn tumor vessels to recruit more blood and nanomedicines into tumors with low metastasis risk.^[^ ^42^ ^]^ CD31 immunofluorescent staining outlined the tumor vascular endothelium and the results showed that PM‐HMSN/Arg + US could most robustly upregulate newborn tumor vessels (Figure 6H and Figure S11A, Supporting Information). As a result of NO‐mediated angiogenesis, improved blood perfusion and oxygen saturation were achieved, alleviating solid tumor hypoxia, and hypoxia‐inducible factor‐1α (HIF‐1α) in the tumor was remarkably downregulated (Figure 6I and Figure S11B, Supporting Information), which is favorable to diminish the hypoxia‐induced immunosuppressive TME. In addition, NO can activate endogenous matrix metalloproteinases (e.g., MMP‐2) to decrease collagen deposition for superior drug delivery,^[^ ^43^ ^]^ and Figure 6J and Figure S11C (Supporting Information) suggested that PM‐HMSN/Arg + US led to the downregulation of type I collagen. Furthermore, activated platelets can accelerate tumor metastasis by promoting epithelial‐mesenchymal transition (EMT) and shedding tumor cells into the blood; therefore, blocking platelet activation can be an appealing approach to retard cancer progression.^[^ ^44^ ^]^ PM‐HMSN/Arg + US treatment resulted in the lowest CD41 expression, indicating a potent inhibition of platelet activation in tumors (Figure 5K and Figure S11D, Supporting Information). Consequently, the antitumor outcomes might be further enhanced by reprogramming the TME into normalization through NO release by PM‐HMSN/Arg.

Taken together, PM‐HMSN/Arg combined with US treatment could effectively suppress tumor via NO induced tumor cells apoptosis and TME modulation.

### 2.6 Biocompatibility

**Figure 7:**
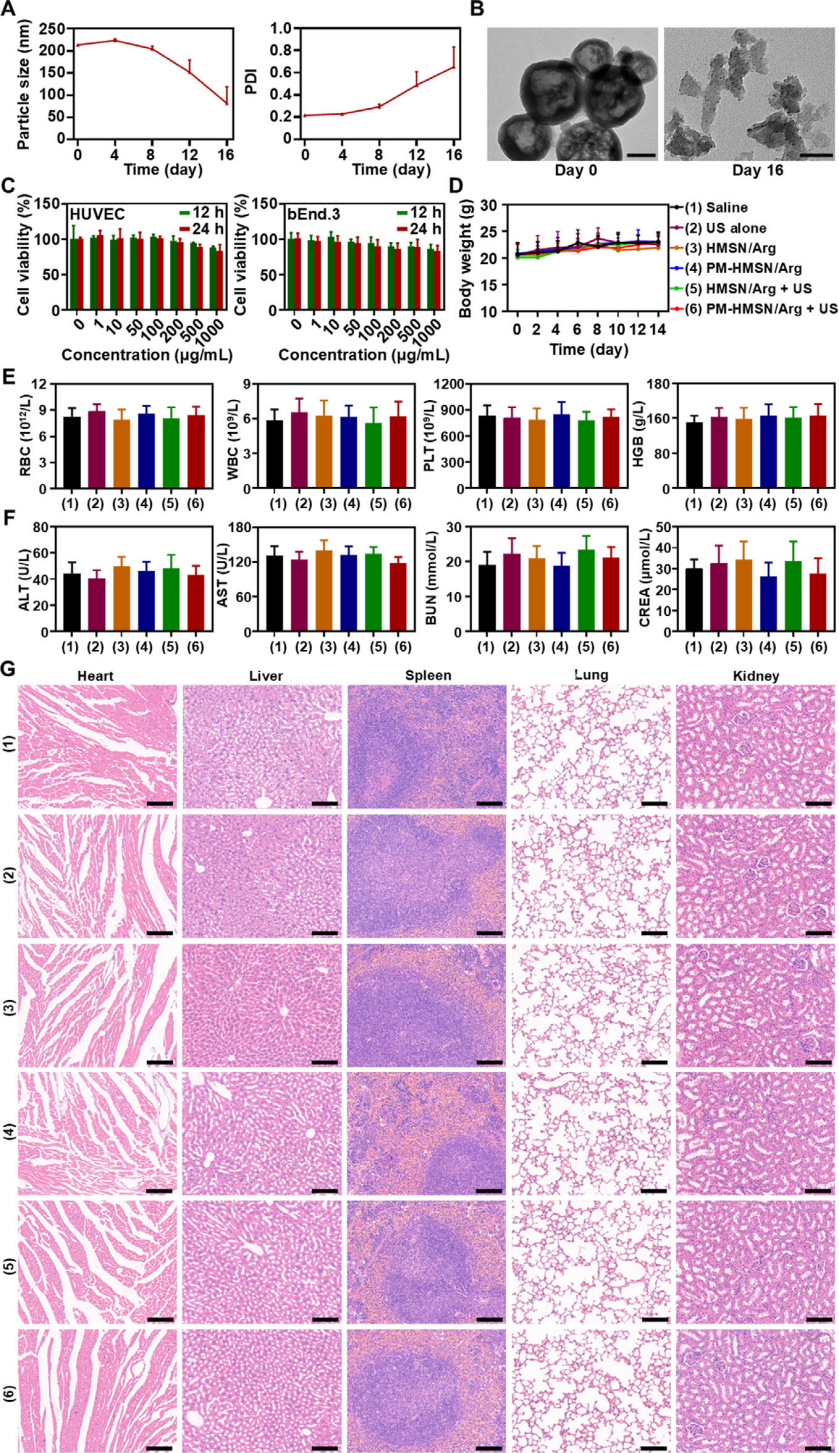
Biosafety of PM‐HMSN/Arg. A) The degradation ability of PM‐HMSN/Arg by monitoring its particle size, polydispersity index (PDI) and B) morphology changes after incubation in plasma at 37 °C at different time intervals. Scale bar: 100 nm. C) In vitro cytotoxicity of PM‐HMSN/Arg assay by determining the cell viability of HUVEC cells and bEnd.3 cells after incubation with PM‐HMSN/Arg at different concentration for 12 and 24 h. Data are presented as mean ± SD (*n* = 3). D) Body weight changes of BxPC‐3 bearing nude mice during NO therapy using various formulations (l‐Arg: 10 mg kg^−1^) with/without US treatment (*n* = 6). E) Blood routine examination and F) blood biochemistry analysis of BxPC‐3 subcutaneous pancreatic tumor model after treatment with the indicated formulations on day 14. Data are presented as mean ± SD (*n* = 3). RBC, red blood cell; WBC, white blood cell; PLT, platelets; HGB, hemoglobin; ALT, alanine aminotransferase; AST, aspartate aminotransferase; BUN, blood urine nitrogen; CREA, serum creatinine. G) Hematoxylin and eosin (H&E) staining of the major organs collected at the end of the treatment. Scale bar: 100 µm.

Considering the possible toxicity of inorganic nanosystem and the crucial role of biosafety of PM‐HMSN/Arg serving as potential cancer theranostic agent for clinical translation, single or multicycle nanosystem treatment were evaluated both in vitro and in vivo. The nanomotor gradually degraded in plasma at physiological temperature, breaking down into tiny nanofragments after 16 days of hydrolysis, suggesting low accumulation toxicity of this inert nanosystem in the body (**Figure** **7**A,B). Then, the cytotoxicity of PM‐HMSN/Arg was evaluated in normal HUVEC cells and bEnd.3 cells based on the reduction activity of methyl thiazolyl tetrazolium (MTT) (Figure 7C). Upon incubation with 1 mg mL^−1^ of PM‐HMSN/Arg, less than 10% of the cells died after a 12 h exposure, and cell viability at 24 h still remained above 80%, suggesting that PM‐HMSN/Arg has relatively low cytotoxicity. Subsequently, the hemolysis test (Figure S12, Supporting Information) showed that PM‐HMSN/Arg in a broad concentration range of 0–5 mg mL^−1^ induced a negligible hemolysis rates of less than 5% upon incubation with erythrocytes, indicating good blood compatibility, which could be attributed to its biocompatible material components and biomimetic PM coating. Moreover, the biosafety of multiple cycles of oncotherapy with PM‐HMSN/Arg with/without US treatment was investigated by determining body weight changes, blood routine examination, biochemistry analysis, and H&E staining of the major organs of the BxPC‐3 tumor‐bearing mice at the end of anticancer therapy (Figure 7D–G). In any of the treatment groups, there was no significant body weight loss or remarkable histological damage in the major organs. Meanwhile, there were no significant differences in various hematology and liver and kidney function indicators between the saline‐treated mice and any other treatment groups, indicating no additional toxicity of long‐term treatment with PM‐HMSN/Arg to blood, liver, and kidneys.

Overall, the biological experiment results supported the favorable biosafety of PM‐HMSN/Arg with good biocompatibility and negligible side effects both in vitro and in vivo.

## 3 Conclusion

In summary, a hollow nanomotor PM‐HMSN/Arg was simply constructed through a layer‐by‐layer coatings method. Upon US activation, PM‐HMSN/Arg produces massive ROS, catalyzing the loaded l‐Arg into NO, which facilitates the relaxation of ECM and active motion in tumor tissue. Consequently, PM‐HMSN/Arg successively overcome multiple bio‐barriers via a distinct “tethering‐relaxing‐drilling” three‐relay cascade delivery mechanism to deeply penetrate cancer post‐administration. Simultaneously, NO can be self‐filled in hollow core as gas supplement for enhanced multimodal theranostics of cancer with an extended lifetime. Given its simple construction, outstanding tumor penetration, distinctly improved bioimaging, and therapy efficacy, this nanomotor represents a promising alternative for precise treatment in low‐penetrable and standard therapies‐resistant cancers, and holds great potential for future clinical translation.

## 4 Experimental Section

### Materials and Reagents

Tetraethyl silicate (TEOS) and cetyl trimethyl ammonium bromide (CTAB) were purchased from Shanghai Aladdin Biochemical Technology Co., Ltd. (Shanghai, China). Nitric oxide assay kit (Griess assay kit) and DAF‐FM DA kit were purchased from Beyotime Institute of Biotechnology (Shanghai, China). l‐Arginine, coumarin‐6 (Cou‐6), 1,1′‐dioctadecyl‐3,3,3′,3′‐tetramethyl indotricarbocyanine iodide (DiR), and ethylenediaminetetraacetic acid (EDTA) were obtained from Dalian Meilun Biotechnology Co., Ltd. (Dalian, China). Hoechst 33342 was purchased from Thermo Fisher Scientific (Eugene, OR). Rabbit anti‐mouse CD41 antibody (ab134131) and CD62P antibody (ab255822) were purchased from Abcam Inc. (Cambridge, MA). Rabbit anti‐mouse CD31 antibody (GB11063‐2), FITC‐conjugated goat anti‐rabbit IgG (GB22303), Cy3‐conjugated goat anti‐rabbit IgG (GB21303), HRP‐conjugated goat anti‐rabbit IgG (GB23303), and FITC‐labeled TUNEL cell apoptosis detection kit were purchased from Servicebio, Inc. (Shanghai, China). RPMI 1640 medium, certified fetal bovine serum (FBS), penicillin‐streptomycin stock solutions, and trypsin‐EDTA (0.25%) were obtained from Gibco (Carlsbad, CA). Other chemicals and reagents were purchased from Sinopharm Chemical Reagent Co. Ltd. (Shanghai, China) unless otherwise mentioned.

### Preparation of PM‐HMSN/Arg–Preparation of HSN

In brief, the water phase (4 g CTAB, 4 g ammonium sulfate, and 56 g distilled water) was adjusted pH ≤ 2 with diluted HCl solution. Then, the ethanol phase (22.8 g ethanol, 10.4 g TEOS) was prepared. After stirring for 0.5 h, the water phase and ethanol phase were mixed for another 0.5 h agitation, followed by 6 h standing. Afterward, the mixture was sprayed by TSI 9302A high‐pressure flow atomizer (20 psi N_2_) and evaporated in high‐temperature tube furnace (400 °C) for inducing the self‐assembly of silicon dioxide. Next, the HSN particles were collected through filter membrane at the outlet, followed by 6 h of calcination at 550 °C to collect the final product HSN.

### Preparation of HMSN

Briefly, the resulted HSN nanoparticles (200 mg) were dispersed in 20 mL of distilled water. Subsequently, 1 mL of NH_3_·H_2_O (40 wt%) was added into 10 mL of distilled water which contains 0.2 mmol MnCl_2_ and 6 mmol NH_4_Cl. Then, the above mixture was poured into the HSN nanoparticles suspension and stirred for 24 h at room temperature, followed by centrifugation at 4000 rpm for 20 min at room temperature. The precipitates at the bottom were collected for freeze‐drying to obtain the final product HMSN.

### Preparation of HMSN/Arg

Briefly, 1 g of l‐Arg was dissolved into 20 mL of deionized water under stirring, and then 100 mg of HMSN was added and stirred for 24 h to sufficiently adsorb l‐Arg molecules, yielding HMSN/Arg. Ultimately, the samples were collected via centrifugation at 4000 rpm for 20 min, followed by freeze‐drying to obtain the HMSN/Arg.

### Preparation of PM‐HMSN/Arg

First, PM was collected according to the preparation procedure in previous study. About 10 mL of whole blood was collected from healthy male BALB/c mice, then the fresh blood samples were centrifuged at 100 and 200 *g*, respectively, for 20 min to remove blood cells. Thereafter, PBS containing 1 × 10^−3^ m EDTA and 2 × 10^−6^ m prostaglandin E1 was added to the supernatant to inhibit platelet activation. The platelets pellets were obtained by centrifugation at 800 *g* for 20 min and were redispersed in 1 mL of PBS, followed by the addition of protease inhibitor tablets and 1 × 10^−3^ m EDTA. Afterward, the harvested platelets were subjected to freeze‐thaw for three repeated cycles. Finally, PM cracks were collected by centrifugation at 4000 *g* for 10 min and dried by lyophilization. Next, PM was suspended in PBS at a concentration of 4 mg mL^−1^ and sonicated repeatedly until the solution became clear and transparent. HMSN/Arg hollow nanoparticles (5 mg mL^−1^) were added into PM suspensions dropwise. The mixture was dispersed repeatedly with a vortex and ultrasound treatment. Then, the above mixture was physically extruded using a mini‐extruder (Avanti), and PM‐HMSN/Arg nanoparticles were finally collected by centrifugation at 4000 rpm for 20 min.

In addition, the fluorescently labeled HMSN by fluorescence probe Cou‐6 or DiR were prepared via solvent evaporation method. Briefly, 1 mg of Cou‐6 or DiR was dissolved in 5 mL of ethanol, then 500 mg of HMSN nanoparticles were added. This mixture was subjected to vacuum drying to remove the ethanol. Afterward, the Cou‐6 or DiR‐labeled HMSN was loaded with l‐Arg or PM just as above. After centrifugation and lyophilization, the fluorescently labeled HMSN/Arg or PM‐HMSN/Arg can be obtained.

### Characterization of PM‐HMSN/Arg–SDS‐PAGE and Western Blot

First, the samples were suspended in deionized water and centrifuged at 10000 rpm for 20 min. Afterward, the precipitates were incubated with pre‐cold RIPA buffer on ice for 30 min to disrupt the intact cell membrane. Next, the protein solution was collected by centrifugation at 15000 *g* for 30 min and the concentrations of extracted protein supernatants were detected by BCA Protein Assay Kit for normalization of protein concentrations prior to mixture with SDS‐PAGE sample loading buffer (Beyotime, China) and boiling for denaturation. Then, the 10% SDS‐polyacrylamide gel was used to separate the proteins by the Novex Xcell Surelock Electrophoresis System (Bio‐Rad, USA), followed by Coomassie brilliant blue protein staining for 6 h and destained with destaining buffer for another 6 h. Finally, the gel was imaged using the Bio‐Rad ChemiDoc Touch Imaging System (Bio‐Rad, USA). In terms of western blot assay, the protein gel was transferred into a poly(vinylidene difluoride) membrane, followed by 5% BSA blocking for 1 h. Subsequently, the membrane was incubated with the antibodies of platelet membrane protein markers anti‐CD41 primary antibody (ab134131) (1:2000 dilution) and anti‐CD62P primary antibody (ab255822) (1:2000 dilution) for 2 h at room temperature. The membrane was washed with TBST solution (Tris‐buffered saline with 0.02% Tween 20) for three times and further incubated with the HRP‐conjugated secondary antibody (goat anti‐rabbit, 1:5000 dilution) for another 2 h. Finally, the blot images were visualized as above.

### BET Analysis

Textural property of HSN nanosphere was measured by N_2_ adsorption–desorption at liquid nitrogen temperature using an Autosorb‐iQ2‐MP (Quantachrome) gas sorption system. Specific surface areas were calculated using the Brunauer–Emmett–Teller (BET) model, meanwhile, the pore size distributions and pore volume were evaluated from the adsorption branches of the nitrogen isotherms using the Barrett–Joyner–Halenda (BJH) model.

### Particle Size and Zeta Potential

The hydrodynamic diameters and zeta potentials of HSN, HMSN, HMSN/Arg, and PM‐HMSN/Arg, respectively, were measured by Malvern Zeta‐sizer Nano ZS90 (Malvern Instruments Ltd., UK). The morphology and structure of HMSN/Arg and PM‐HMSN/Arg were studied by TEM (JEOL, JEM‐2000EX, Japan) conduced at 120 keV.

### l‐Arg Loading Capacity Determination

The loading amount of l‐Arg in nanospheres was measured by a commercially available l‐Arg ELISA kit (General L‐Arg ELISA Kit, EIAab, Wuhan, China) according to the manufacturer's protocols.

### TEM Analysis

TEM (FEI Tecnai 30, 300 kV, Philips) was used for morphology observation of HMSN/Arg and PM‐HMSN/Arg.

### Element Mappings Assay

TF‐G20 high‐resolution TEM (Thermo Fisher Scientific, USA) was adopted to characterize PM‐HMSN/Arg morphology and analyzed the distribution of Si, O, Mn, N, and P.

### FTIR Analysis

FTIR spectra were recorded on a Tensor‐II FTIR spectrometer (Bruker Co., Germany) by averaging 64 scans with a spatial resolution of 0.5 cm^−1^. The tested samples were used KBr pellets containing l‐Arg, HSN, HMSN, HMSN/Arg, and PM‐ HMSN/Arg, respectively.

### Stability Assay

The physical stability of PM‐HMSN/Arg during storage was evaluated by observing the DLS size changes. Briefly, PM‐HMSN/Arg was suspended in 10% FBS solutions at 37 °C. At various time intervals (0, 2, 4, 6, 12, 24, 48, and 72 h), the samples were collected to analyze the size changes of nanoparticles. In addition, the mechanical stability of PM‐HMSN/Arg toward the sonication was also investigated. Briefly, the PM‐HMSN/Arg samples were dispersed in 10% FBS solution and then subjected the US irradiation for 1–6 cycles. The US condition per cycle was set as 1.0 MHz, 1.5 W cm^−2^, 15 min, the duration time of each pulse was 15 s with a 30 s interval between two adjacent pulses, and the time interval between each cycle was 1 h. Simultaneously, the particle size was recorded at each timepoint after US treatment.

### ROS Generation Assay–Quantitative Analysis of the Generated ^1^O_2_

Briefly, 200 µg mL^−1^ of 9,10‐diphenanthraquinone (DPA) was mixed with PM‐HSN, PM‐HMSN, or PM‐HMSN/Arg suspensions (the nanoparticles amount all equal to ≈100 µg mL^−1^ of HSN), respectively, at a volume ratio of 40:1. Next, the degradation curve of DPA by different nanoparticles under different US irradiation (1.0 MHz, 1.5 W cm^−2^) durations was recorded with UV–vis spectrophotometer. DJO‐2776 sonicator as an energy converter was applied to generate ultrasound during the treatment. Meanwhile, the US alone treated DAP solution without nanoparticles, and the mixture of HSN, HMSN, or HMSN/Arg, respectively, with DPA solution but without US treatment were performed according to the same protocol as above for control.

### Quantitative Analysis of the Generated ·OH

250 µg of methylene blue (MB) was added to 45 mL of PBS, followed by the introduction of PM‐HSN, PM‐HMSN, or PM‐HMSN/Arg suspensions (the nanoparticles mass all equal to 5 mg of HSN). With different US (1.0 MHz, 1.5 W cm^−2^) irradiation durations, the MB was degraded by the ·OH generated in these nanoparticles. Accordingly, the degradation curve was recorded by UV–vis spectrophotometer. In addition, the US alone treated MB solution without nanoparticles, and the mixture of PM‐HSN, PM‐HMSN, or PM‐HMSN/Arg, respectively, with MB solution but without US irradiation were operated as above to serve as control group.

### Propulsion Performance of Nanomotor PM‐HMSN/Arg

The movement of micromotors were recorded using Nanoparticle Tracking Analysis (NanoSight Pro, Malvern). All the experiments were performed in PBS solution by introducing different samples at the same concentration. Meanwhile, DJO‐2776 sonicator was used for providing ultrasound pretreatment (1.0 MHz, 1.5 W cm^−2^), followed by immediate trajectories tracing. According to the X–Y coordinates of the micromotor trajectories, captured from the optical tracking, the MSD was calculated by the following equation, and 20 nanomotors were used for each condition:

(1) $$\mathrm{MSD}\left(\Delta t\right)=<\frac{1}{N}\sum_{i=1}^{N}{\left|\vec{r_{i}}\left(t_{0}+\Delta t\right)-\vec{r_{i}}\left(t_{0}\right)\right|}^{2}>$$

### In Vitro US/T1‐MRI Dual‐Modality Imaging

The ultrasound imaging ability of PM‐HMSN/Arg was investigated by the 2D grayscale ultrasound and contrast‐enhanced ultrasonography (CEUS) model on the SonoScape scanner equipped with a 1 MHz convex array probe at a mechanical index (MI) of 0.16. Briefly, 1 mg mL^−1^ of PM‐HMSN, PM‐HSN/Arg, and PM‐HMSN/Arg suspensions diluted in saline, respectively, was filled in the centrifuge tube and subjected to the ultrasonic scanner probe guided scanning. Meanwhile, the physiological saline solution without nanoparticles was performed according to the same proceedings to serve as control. The US images at different timepoints were recorded. All the measurements were conducted at room temperature.

The T1‐weighted MRI signals of PM‐HMSN/Arg were measured by Bruker Biospect 7.0T/20cm magnetic resonance system at room temperature (TE = 50 ms, TR = 3000 ms). For in vitro MRI of PM‐HMSN/Arg, the prepared PM‐HMSN/Arg was dispersed into PBS with different pH of 7.4 or 5.5, respectively. Then, the PM‐HMSN/Arg solution was diluted to corresponding Mn concentrations (0, 0.05, 0.1, 0.2, 0.5, 1 × 10^−3^ m) for in vitro T1‐MRI testing in quartz nuclear magnetic tube and analyzed using Paravison 5.0 software.

### Penetration Assay of PM‐HMSN/Arg within 3D Tumor Spheroids

To establish B&N hybrid 3D tumor spheroids, BxPC‐3 and NIH3T3 cells at a ratio of 1:3 were seeded onto 96‐well plates that were precoated with 2.0% agarose solution (w/v). Subsequently, the cells were cultured for 7 d to grow into spheroids, followed by the treatment with 10 ng mL^−1^ TGF‐β to activate NIH3T3 cells for abundant stroma synthesis and secretion to simulate the tumor penetration barrier. Afterward, the spheroids were incubated with Cou‐6 labeled PM‐HMSN/Arg (l‐Arg: 1 mg mL^−1^) with or without US treatment with the condition set as 1.0 MHz, 1.5 W cm^−2^, 15 min, the duration time of each pulse was 15 s with a 30 s interval between two adjacent pulses. Finally, the fluorescence intensity and distribution within tumor spheroids were imaged under a confocal laser scanning microscopy (CLSM) (Leica TCS SP8 STED, Leica, Germany).

### Tumor Perfusion Assay

The measurement of whole and functional vessels in tumors was adopted here to estimate the tumor perfusion level after different treatments. Briefly, the mice with the tumor volume around 100 mm^3^ were randomly divided into six groups. Group 1: saline; Group 2: US irradiation; Group 3: HMSN/Arg; Group 4: PM‐HMSN/Arg; Group 5: HMSN/Arg + US irradiation; Group 6: PM‐HMSN/Arg + US irradiation. The mice of each group were injected through tail vein with varied formulations at 100 µL, and the content of l‐Arg was controlled at 10 mg kg^−1^. The US irradiation referred to that the tumor tissues were radiated with US on portable focused ultrasound therapeutic apparatus DJO‐2776 sonicator, and the parameter of each cycle was 1.0 MHz‐1.0 W‐20% for 15 s with a 30 s interval between two cycles, and 20 cycles of US irradiation were carried out. The irradiations started at 15 min after varied formulations injection. After the US treatment, the mice were injected with FITC‐labeled lectin (about 0.1 mg per mice). After another 20 min post‐injection, the mice were sacrificed to remove the tumors for cryo‐sectioning. Afterward, the sections were subjected to immunofluorescence staining with anti‐CD31 antibody to label tumor vascular endothelial cells, followed by CLSM imaging. The percentage of double‐positive lectin and CD31 functional vessels was evaluated using ImageJ software.

### Hemolysis Assay

The hemolysis assay was performed according to the previously reported method with minor modification. Briefly, red blood cells (RBCs) were isolated from fresh heparinized mouse blood and washed with PBS three times via three centrifugation cycles at 2000 rpm for 5 min at 4 °C until the supernatant was clear. PM‐HMSN/Arg at different concentration (0–1 mg mL^−1^) were added into 2% (v/v) RBC suspension and incubated at 37 °C for 2 h. Then, these incubation solutions were centrifuged at 2000 rpm for 5 min to separate the supernatant from the RBCs. The absorbance of supernatant was monitored at 570 nm with a microplate reader (Multiskan MK3, Thermo Fisher Scientific, USA). The hemolysis rate was calculated using the following equation:

(2) $$\mathrm{Hemolysis}\;\mathrm{rate}\;\left(\%\right)=\left[\frac{\left(OD_{\mathrm{test}\;}-\;OD_{\mathrm{neg}}\right)}{\left(OD_{\mathrm{pos}\;}-\;OD_{\mathrm{neg}}\right)}\right]\times \;100$$

where OD_test_, OD_neg_, and OD_pos_ were the OD_570_ values of samples, negative control (saline), and positive control (deionized water), respectively.

Meanwhile, the images of the supernatants were taken using a digital camera. While the morphological images of the RBCs were captured using a microscope (Leica DMI 4000D, Germany).

### Cytotoxicity Assay

HUVEC cells or bEnd.3 cells were seeded in 96‐well cell culture plate at 5 × 10^4^ cm^−2^ density and cultured at 37 °C for 24 h. The cells were treated with PBS or PM‐HSMN/Arg at a series of concentrations (0, 1, 10, 50, 100, 200, 500, and 1000 µg mL^−1^) at 37 °C for 12 or 24 h, respectively. Subsequently, 10 µL of MTT stock solution (5 mg mL^−1^) was introduced into the culture medium for another 4 h incubation. Then, the culture medium was discarded and 200 µL of DMSO was added to dissolve the formazan in each well. Finally, the absorbance at 570 nm were measured by microplate reader (Synergy 2, Bio‐tek, USA).

### Biosafety Assay

To evaluate the degradation process of PM‐HMSN/Arg, the nanomotors (1 mg mL^−1^) were incubated with mice plasma to simulate the in vivo microenvironment, followed by the corresponding particle size and PDI monitoring of PM‐HMSN/Arg every day for 16 d, and the particle morphology change was also observed by TEM.

In terms of the in vivo biosafety, the blood samples were drawn at the end of antitumor therapy, followed by the blood routine examination and blood biochemistry analysis. Meanwhile, the major organs including heart, liver, spleen, lungs, and kidneys were collected for H&E staining.

### Cell Lines and Animals

The human pancreatic cancer cell line BxPC‐3 and the HUVEC were provided by the Cell Bank of the Chinese Academy of Sciences (Shanghai, China). BxPC‐3 and HUVEC cells were cultured at 37 °C under 5% CO_2_ in RPMI 1640 supplemented with 10% FBS and 1% penicillin/streptomycin.

Male Balb/c nude mice (6–8 weeks) were supplied by Shanghai SLAC Laboratory Animal Co., Ltd. (China) and maintained under specific‐pathogen‐free laboratory conditions in the Experimental Animal Center, School of Medicine, Wuhan University of Science and Technology.

### In Vitro NO Release Assay of PM‐HMSN/Arg

The NO release profile in vitro was investigated using a Griess assay kit. Briefly, PM‐HMSN, PM‐HSN/Arg, and PM‐HMSN/Arg, were added with the same equivalent to 50 mL of PBS (pH 7.4). In particular, for PM‐HSN/Arg and PM‐HMSN/Arg, they contain 5 mg of l‐Arg. Then, the US treatment was carried out under the conditions of 1.0 MHz, 1.5 W cm^−2^, for 30 min. Each pulse consisted of an alternating 15 s on and 30 s off cycle. Meanwhile, the temperature of the solutions was maintained at 37 °C during the US treatment. After that, 1 mL of dispersion was collected and replaced with 1 mL of fresh PBS at predetermined time points (0, 5, 10, 15, 20, 25, and 30 min). The dispersion was centrifuged at 4000 rpm for 5 min, and the supernatant was used for NO determination via the Griess assay kit. In addition, the NO generation from PM‐HMSN, PM‐HSN/Arg, and PM‐HMSN/Arg without US treatment was determined using the same protocol to serve as controls.

### Targeting Effect of PM‐HMSN on HUVEC and BxPC‐3 Cells

HUVEC cells were treated with VEGF (10 nmol) and cultured for 7 d to establish a cell model of tumor vascular endothelium as previously reported.^[^ ^33^ ^]^ Then, the HUVEC cells were seeded in a glass‐bottom 24‐well plate at a cell density of 1 × 10^5^ cells per well. After culturing for 24 h, the medium containing fluorescent Cou‐6‐labeled HMSN or PM‐HMSN was added to replace the original cell culture medium. Considering the rapid binding of PM with membrane ligands on HUVEC cells and the short contacting time of nanomotors with tumor vessel walls in vivo, the incubation time was set to 0.5 h. Then, the culture medium was discarded and the cells were washed with PBS several times. Afterward, the cells were stained with Hoechst 33342 to label the nuclei, followed by CLSM (Leica TCS SP8 STED, Leica, Germany) fluorescence imaging. In a parallel operation as aforementioned, the cell samples after incubation with nanoparticles were digested and resuspended, followed by flow cytometry analysis (CytoFLEXS, Beckman Coulter, USA, λ_ex_ = 488 nm, λ_em_ = 525 nm). Similarly, BxPC‐3 cells were also seeded in a glass‐bottom 24‐well plate at the same density and cultured as described above. Given that the nanomotors can be retained within tumor tissues to adequately interact with tumor cells, the incubation time of BxPC‐3 cells with Cou‐6‐labeled HMSN or PM‐HMSN was set to 2 h. Then, the cells were stained with Hoechst 33342, followed by CLSM imaging and flow cytometry analysis as described above.

### Intracellular NO Content Assay

DAF‐FM DA was also used to evaluate the intracellular NO content. Briefly, BxPC‐3 cells were seeded in a 24‐well glass‐bottom cell‐culture plate at a density of 2 × 10^5^ cells per well. Then, the BxPC‐3 cells were pretreated with NO fluorescent probe DAF‐FM DA (1 × 10^−6^ m) in the dark for 20 min to achieve in situ loading of DAF‐FM DA. Afterward, the probes were discarded and the tumor cells were washed with PBS several times. Next, the cells were incubated with HMSN/Arg or PM‐HMSN/Arg (l‐Arg: 0.1 mg mL^−1^), and subjected to US treatment under the condition of 1.0 MHz, 1.5 W cm^−2^, for 15 min. Each pulse consisted of an alternating 15 s on and 30 s off cycle, with the incubation temperature at 37 °C. After 30 min of incubation, the cells were fixed with 4% paraformaldehyde and stained with Hoechst 33342 to label the nuclei. Finally, the fluorescence intensity of DAF‐FM DA in the cells was imaged under a CLSM (Leica TCS SP8 STED, Leica, Germany). Besides, NO contents of BxPC‐3 cells treated with PBS, HMSN/Arg, and PM‐ HMSN/Arg without US irradiation were determined in the same protocol to serve as the control.

### NO‐Induced Antitumor Effect In Vitro

BxPC‐3 cells were seeded in a 24‐well glass‐bottom cell‐culture plate at the aforementioned cell density and were cultured in 5% CO_2_ at 37 °C for 24 h. Next, BxPC‐3 cells were incubated with PBS, HMSN/Arg, or PM‐HMSN/Arg (l‐Arg: 0.1 mg mL^−1^), respectively, followed by US irradiation with the aforementioned conditions for 30 min. After another 24 h incubation, the cells were stained with a Calcein‐AM/PI probe at a concentration of 4 mmol L^−1^ at 37 °C for 15 min, followed by PBS washing three times and CLSM observation. In addition, ImageJ software was used for fluorescence semi‐quantification to compare the proportion of living and dead cells. For cytotoxicity evaluation, the same procedures were followed.

### Establishment of Subcutaneous BxPC‐3 Bearing Nude Mice Model

All animal experiments were conducted following the guidelines approved by the Institutional Animal Care and Use Committee (IACUC) of the School of Medicine, Wuhan University of Science and Technology. The murine pancreatic cancer model was established by inoculating 8 × 10^6^ BxPC‐3 cells into the subcutaneous tissue of the right forelimbs of nude mice.

### In Vivo Distribution of PM‐HMSN/Arg

The distribution of PM‐HMSN/Arg in vivo was assessed via fluorescence imaging using an IVIS Spectrum Imaging System (PerkinElmer, USA). When the tumor volume reached ≈100 mm^3^, the BxPC‐3 tumor‐bearing mice were intravenously injected with HMSN/Arg or PM‐HMSN/Arg, respectively (DiR: 0.5 mg kg^−1^). At 15 min post‐administration, the tumor tissues were irradiated with US on a portable focused ultrasound therapeutic apparatus DJO‐2776 sonicator, with the parameters of 1.0 MHz‐1.0 W‐20% for 15 s with a 30 s interval between cycles, and 20 cycles of US irradiation per round. No significant US or heat damage was observed in the mice. Meanwhile, tumor‐bearing mice treated with nanomotors but without US irradiation were set as controls. Subsequently, the mice were anesthetized by inhalation of isoflurane and photographed using an in vivo IVIS spectrum imaging system at predetermined intervals (15 min, 6 h, and 24 h) (PerkinElmer, USA). The mice were then anesthetized, perfused with saline, and sacrificed. The tumors and major organs were collected for ex vivo fluorescence imaging.

### Intratumor Penetration of PM‐HMSN/Arg

The intratumor permeability of PM‐HMSN/Arg was evaluated via labeling particles with Cou‐6. Briefly, the mice with a tumor volume of ≈100 mm^3^ were intravenously administrated with Cou‐6‐labeled HMSN/Arg or PM‐HMSN/Arg, respectively (Cou‐6: 0.5 mg kg^−1^), followed by US treatment at 15 min post‐administration as illustrated in biodistribution assay. At predetermined time points (15 min and 6 h), the mice were anesthetized, followed by perfusion with saline and 4% paraformaldehyde. After that, the tumors were sliced and incubated with anti‐CD31 antibody (1:200) overnight at 4°C, followed by staining with Cy3‐conjugated goat anti‐rabbit IgG GB21303 (1:500 dilution) for 2 h at 37°C to label the tumor vessels. The cell nuclei were counterstained with DAPI. Finally, the tumor slices were visualized under CLSM.

### Intratumor NO Release Assay

The intratumor NO release of PM‐HMSN/Arg was assayed using the NO probe DAF‐FM DA. The BxPC‐3 subcutaneous tumor‐bearing mice with a tumor volume around 100 mm^3^ were randomly divided into six groups (*n* = 6). Group 1: saline; Group 2: US irradiation; Group 3: HMSN/Arg; Group 4: PM‐HMSN/Arg; Group 5: HMSN/Arg + US irradiation; Group 6: PM‐HMSN/Arg + US irradiation. The mice of each group were injected through the tail vein with varied formulations at 100 µL, and the content of l‐Arg was controlled at 10 mg kg^−1^. Before the test, the mice were intratumorally injected with DAF‐FM DA (2.5 mg kg^−1^) to monitor the NO level within the tumors. Besides, groups with US irradiation referred to that the tumor tissues were irradiated with US using a portable focused ultrasound therapeutic apparatus DJO‐2776 sonicator with the same parameters as the above experiments. After the US treatment, the tumors were collected for cryosection, and slices were stained with Hoechst 33258 for CLSM observation.

### In Vivo US/T1‐MRI Dual‐Modality Imaging

For in vivo US imaging, BxPC‐3 tumor‐bearing mice with a volume of around 100 mm^3^ were intravenously injected with the same dose of PM‐HMSN, HMSN/Arg, and PM‐HMSN/Arg, respectively (l‐Arg: 10 mg kg^−1^). Then, the US images were recorded at different time points (2 min, 10 min, 30 min, 1 h, and 2 h).

For in vivo T1‐MRI imaging of PM‐HMSN/Arg, BxPC‐3 tumor‐bearing mice with a volume of around 100 mm^3^ were intravenously injected with the same dose of HMSN/Arg or PM‐HMSN/Arg, respectively (l‐Arg: 10 mg kg^−1^). After the same US treatment as in the in vivo distribution experiment, T1‐MRI images were recorded at different time points (2 and 12 h). In addition, the samples without US irradiation were also recorded as controls.

### Antitumor Effect Assay in BxPC‐3 Xenograft Pancreatic Cancer Model

The antitumor efficacy of PM‐HMSN/Arg was evaluated in a BxPC‐3 subcutaneous xenograft model. The tumor‐bearing mice with a tumor volume of around 100 mm^3^ were randomly divided into six groups (*n* = 6). Group 1: saline; Group 2: US irradiation; Group 3: HMSN/Arg; Group 4: PM‐HMSN/Arg; Group 5: HMSN/Arg + US irradiation; Group 6: PM‐HMSN/Arg + US irradiation. The mice in each group were injected through the tail vein with varied formulations at 100 µL, and the content of L‐Arg was controlled at 10 mg kg^−1^. US treatment was performed every 2 d for seven cycles. The tumor diameter and body weight of the mice were recorded every 2 d. The tumor volume (*V*) was calculated using the equation:

(3) $$V=\frac{1}{2}\;ab^{2}$$

where *a* and *b* represent the long and short diameters of the tumors, respectively.

At the end of the therapy, the mice were sacrificed to harvest the organs and tumors, followed by weighing and staining with H&E and TUNEL to evaluate the apoptosis levels using the following equation:

(4) $$\mathrm{Apoptosis}\;\mathrm{index}\;\left(\%\right)=\frac{A_{\mathrm{TUNEL}}}{A_{\mathrm{DAPI}}}\;\times 100\%$$

where *A*_DAPI_ and *A*_TUNEL_ represent the numbers of total cells and the apoptotic cells stained by DAPI and TUNEL, respectively, counted from five random fields of view.

The IRT (%) was calculated using the equation as follows:

(5) $$\mathrm{IRT}\;\left(\%\right)=\frac{\left(W_{\mathrm{control}}-\;W_{\mathrm{treated}}\right)}{W_{\mathrm{control}}}\;\times \;100\%$$

where *W*_control_ and *W*_treated_ indicate the average tumor weight in the control and treatment groups, respectively.

Finally, the tumor tissues were sliced for immune‐staining of CD31, collagen, HIF‐1α, and CD41, respectively.

### Statistical Analysis

The results are displayed as mean ± standard deviation (SD). An independent‐sample *t*‐test was applied to evaluate the significant differences, except that multiple treatment groups were compared within individual tests by nonparametric two‐tailed analysis of variance (ANOVA) followed by a Tukey's post hoc test. In each case, *p* ≤ 0.05 was considered statistically significant.

## Conflict of Interest

The authors declare no conflict of interest.

## Author Contributions

X.X., J.C., and Y.M. are co‐authors and contributed equally to this work. X.X. and Q.H.: conceptualization; J.C. and M.C.: methodology; X.X., J.C. and M.C.: investigation; Y.L. and J.C.: writing – original draft; Y.M., H.Z., and Y.W.: writing – review and editing; X.X.: funding acquisition; Q.H., and Y.L.: supervision.

[Correction added on 25 June 2025, after first online publication: equal contribution statement is added.]

## Supporting information



Supporting Information

Supporting Information

Supporting Information

Supporting Information

Supporting Information

Supporting Information

Supporting Information

## Data Availability

The data that support the findings of this study are available in the supplementary material of this article.
