# Supplementary material for: Ultrasound‐Induced Nitric Oxide‐Propelled Nanomotor for Multimodal Theranostics of Cancer with Deep Penetration and Extended Lifetime
Source: Adv Sci (Weinh). 2025 Jun 10;12(30):e16709. doi: 10.1002/advs.202416709 (PMC12376693; doi:10.1002/advs.202416709)
Supplement: Supplementary file 1 — Supporting Information [file ADVS-12-e16709-s006.docx]

Supporting Information for

**Ultrasound-Induced Nitric Oxide-****Propelled Nanomotor for** **Multimodal** **Theranostics of Cancer with** **Deep Penetration and** **Extended Lifetime**

Xue Xu^a,#^, Jinxu Cao^a,#^, Yan Mu^b,#^, Hao Zhang^b^, Ya-Lei Wang^b^, Mengzhen Cheng^a^, Yuce Li^c,^*, Qian Hua^b,^*

^a^ School of Medicine, Wuhan University of Science and Technology, Wuhan 430065, China

^b^ School of Life Sciences, School of Chinese Medicine, Beijing University of Chinese medicine, Beijing, 100029

^c^ College of Life Sciences and Health, Wuhan University of Science and Technology, Wuhan 430065, China

**^#^ Contributing equally**

***Corresponding authors**

Prof. Y. Li. E-mail: liyuce@wust.edu.cn

Prof. Q. Hua. E-mail: huaq@bucm.edu.cn

**1. Supplementary Data and Figures**

Firstly, HSN with typical mesoporous features of large surface area (508.18 m^2^g^−1^), pore volume (0.403 cm^3^g^−1^) and pore size (4.3 nm), was synthesized by aerosol-induced self-assembly method (**Fig. S****1)** ^[1]^. After layer-by-layer coatings of Mn, L-Arg and platelet membrane (PM), respectively, the HSN, HMSN, HMSN/Arg and PM-HMSN/Arg nanosphere showed a uniform size distribution of 192.3 nm, 194.6 nm, 196.8 nm and 215.4 nm, respectively (**Fig. S2**).

The stability of PM-HMSN/Arg was evaluated via observing its particle size changes in 10% FBS (**Fig. S4 and 5**), and no apparent diameter change after incubation within 72 h or multiple cycles of US irradiation, suggesting its good stability.

Next, we monitored the ^1^O_2_ and •OH (as the main ROS) generation capability by employing 9,10-diphenanthraquinone (DPA) and methylene blue (MB) chemical probe and measuring their absorbance changes in different nanosphere suspensions ^[2]^. In **Fig. S6 and 7**, without US treatment, no apparent absorbance decreases of DPA and MB can be observed, suggesting negligible ^1^O_2_ and •OH yielding. On the contrary, once the US radiation was carried out, the characteristic absorption peak intensity of DPA and MB decreased rapidly within several minutes. In detail, the decomposition rates of each group were ranked as follows: US alone < PM-HMSN/Arg+ US < PM-HSN+ US < PM-HMSN+ US, indicating the effective ROS generation in US triggered nanospheres. Additionally, the above trend can be explained as follows: (1) the mesoporous silicate nanosphere could provide the platform for energy transformation from US mechanical energy to chemical energy of ROS ^[2]^; (2) Mn element could promote ROS yielding of nanosphere because low valence Mn can be partially oxidized by holes to high valence, which accelerated the separation of electron-holes ^[3]^; (3) ROS can rapidly oxidize L-Arg into NO, leading to the remarkable consumption of ROS.

Subsequently, the Mn ions usually possess positive enhancing effect on T1-MRI signal ^[4]^, thereby we anticipated a T1-weighted MRI capability of Mn-containing PM-HMSN/Arg. To prove this hypothesis, the *in vitro* T1-weighted MRI of PM-HSN/Arg incubated in phosphate buffer solution (PBS) at different pH was investigated because the pH of the tumor extracellular microenvironment is about 7.2~6.5 while that of the intracellular endosome and lysosome reaches to 6.2~5.0 ^[5]^. As shown in **Fig. S8**, with the Mn concentration increased, bright T1-weighted MRI signal enhancements could be observed in both pH 7.4 and pH 5.5 PBS, showing a Mn concentration-dependent T1-MRI imaging. Moreover, the signal in pH 5.5 PBS increased much faster than that in pH 7.4 PBS, and the relaxation rate (r_1_ value) of PM-HSN/Arg at pH 7.4 was measured to be only 0.428 mM^-1^ s^-1^, and it increased to 5.860 mM^-1^ s^-1^at pH 5.5, displaying a pH sensitive T1-MRI capability. This result could be ascribed to the greater dissociation of Mn ions in aqueous environment under tumor acid condition as reported before ^[6]^.

**
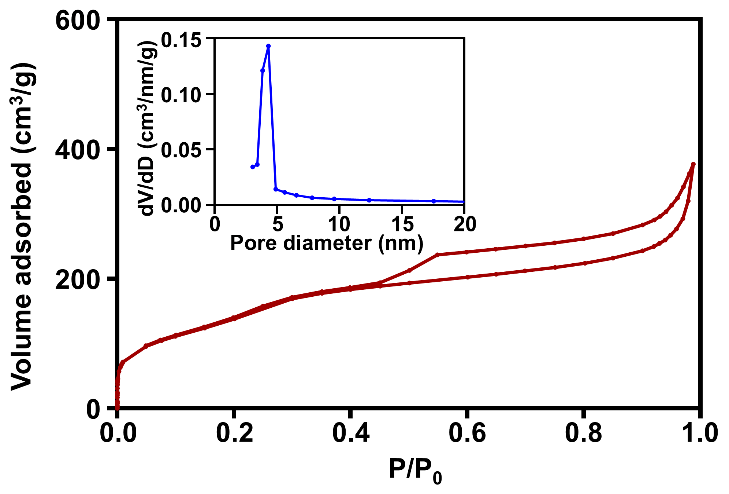
**

**Fig. S1.** N_2_ adsorption-desorption isotherm corresponding pore size distribution curve of HSN nanosphere.

**
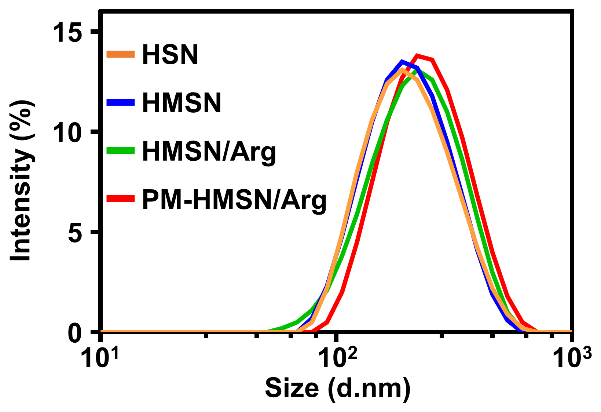
**

**Fig. S2.** Particle size distribution of different nanomotors.

**
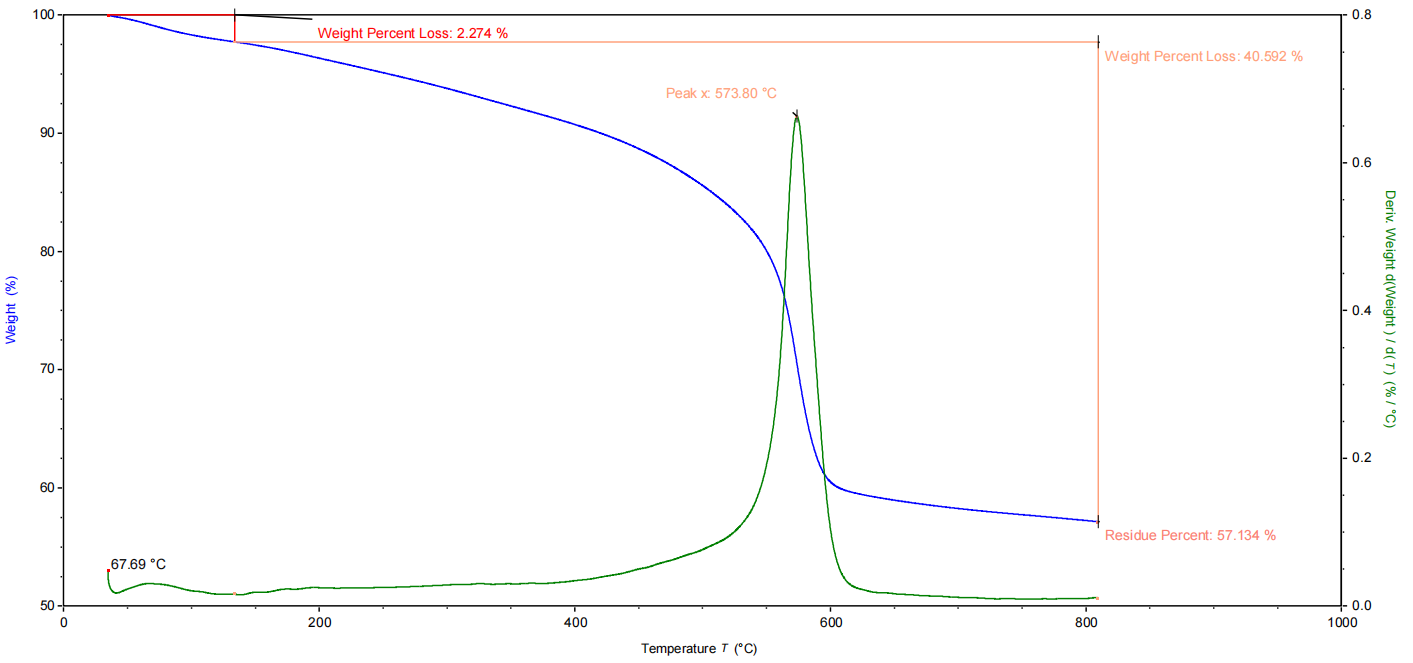
**

**Fig. S3.** Thermogravimetric analysis of PM-HMSN.


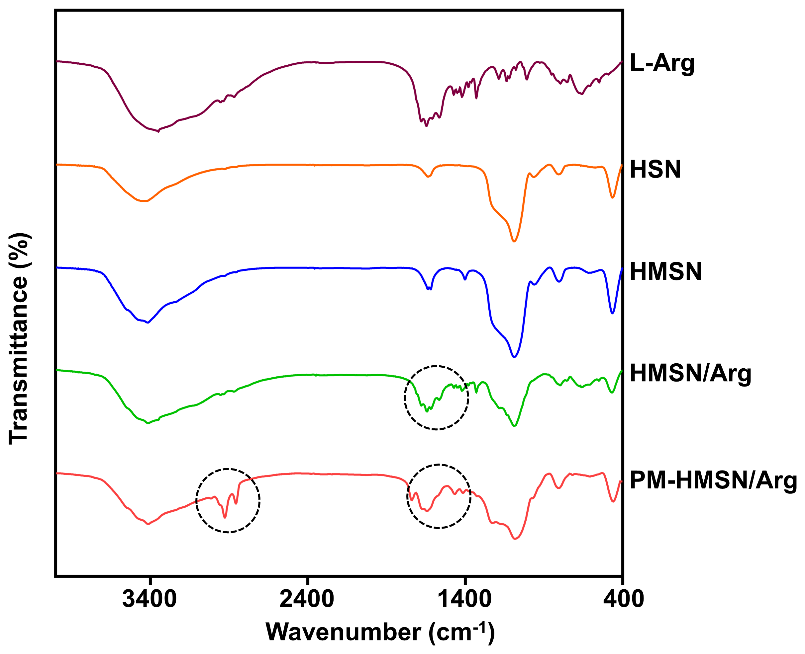


**Fig. S4.** FTIR spectra for L-Arg, HSN, HMSN, HMSN/Arg and PM-HMSN/Arg: ranging from 4000 cm^-1^ to 400 cm^-1^.


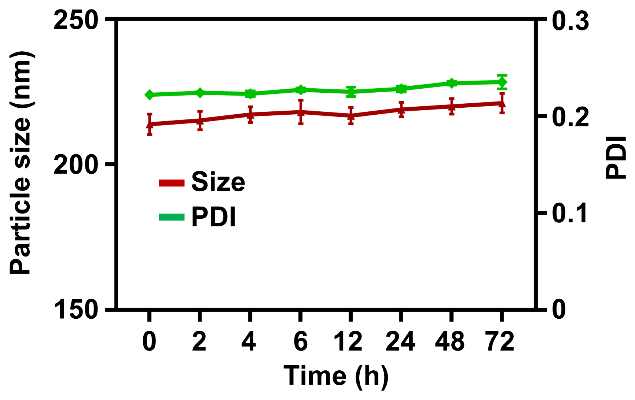


**Fig. S5.** The physical stability of PM-HMSN/Arg via determining the particle size and polydispersity index (PDI) changes after incubation in 10% FBS solution at 37 ℃ for different time intervals (n = 3).


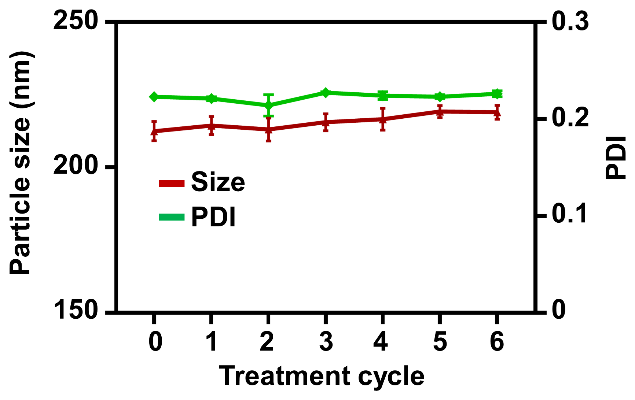


**Fig. S6.** The mechanical stability of PM-HMSN/Arg towards the sonication with different US treatment cycles in 10% FBS solution (n = 3).


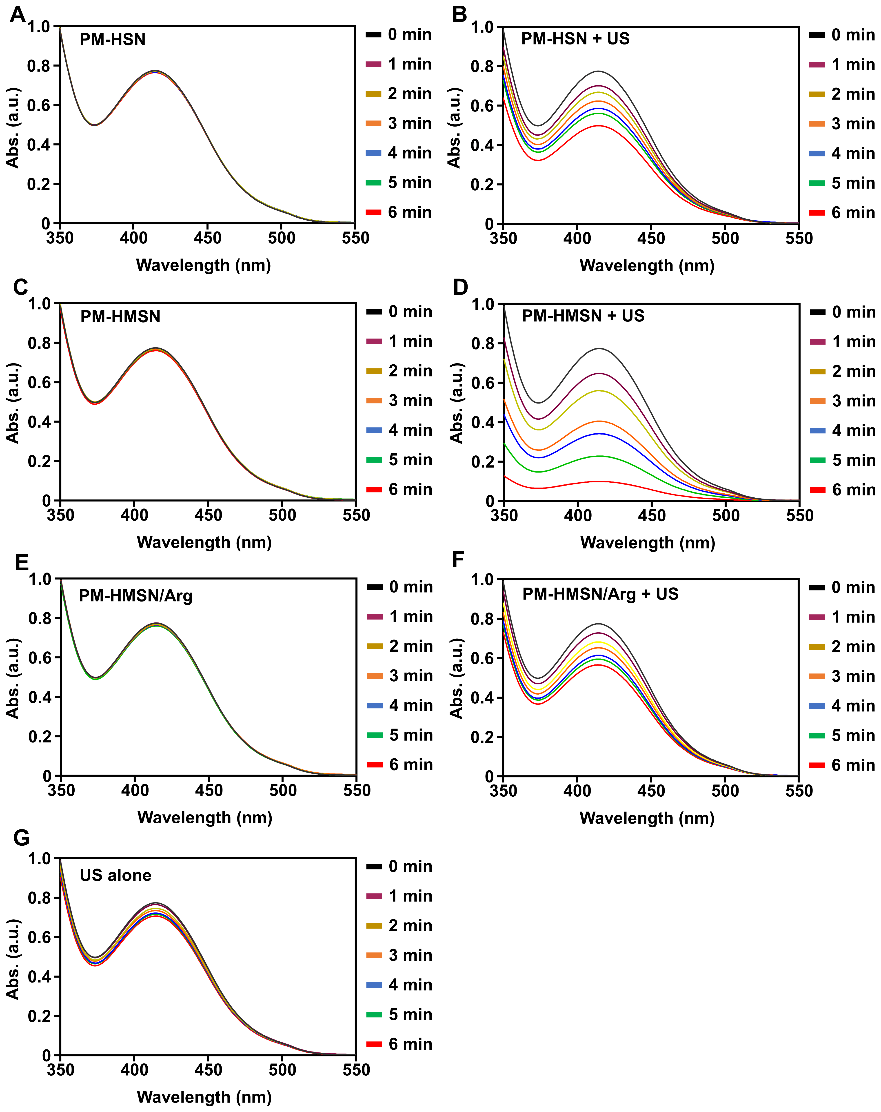


**Fig. S7.** The time-dependent degradation of DPA treated with different formulation with/without US (1.0 MHz, 1.5 Wcm^−2^) irradiation for ^1^O_2_ yielding assay.

**
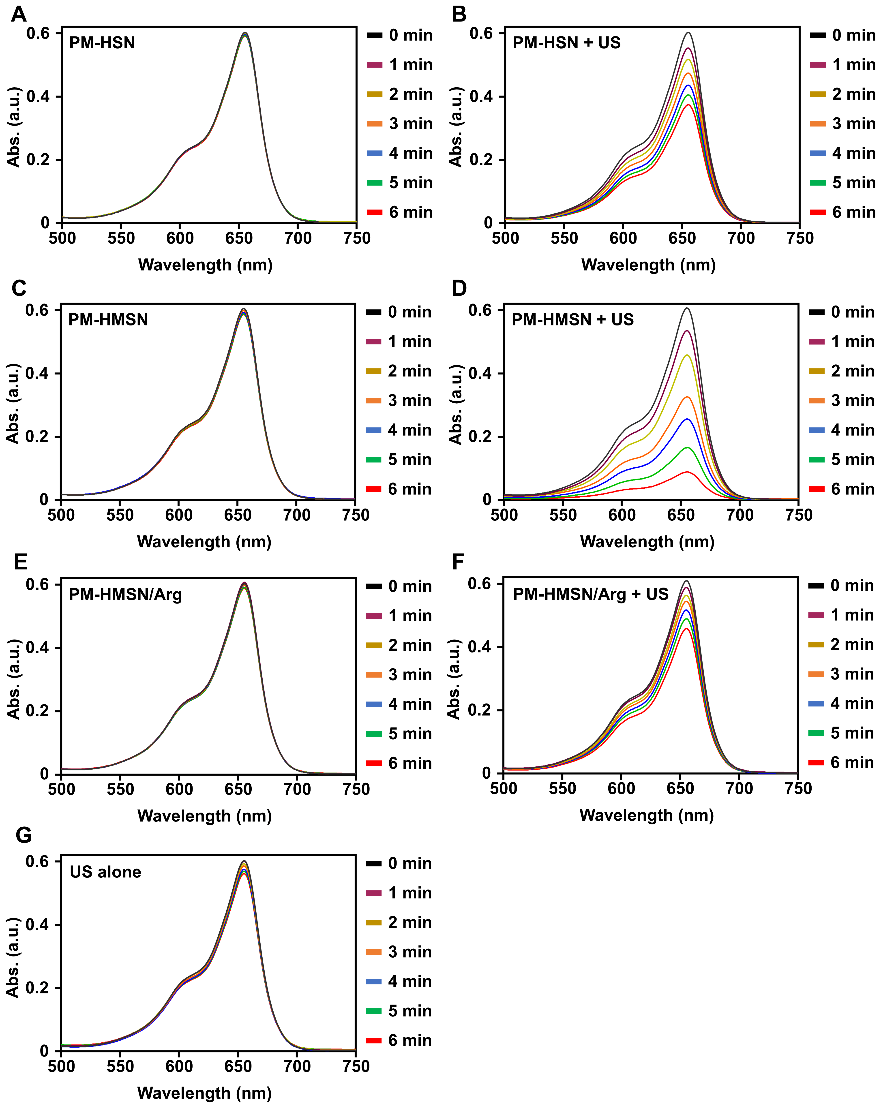
**

**Fig. S8.** The time-dependent degradation of MB treated with different formulation with/without US (1.0 MHz, 1.5 Wcm^−2^) irradiation for •OH yielding assay.


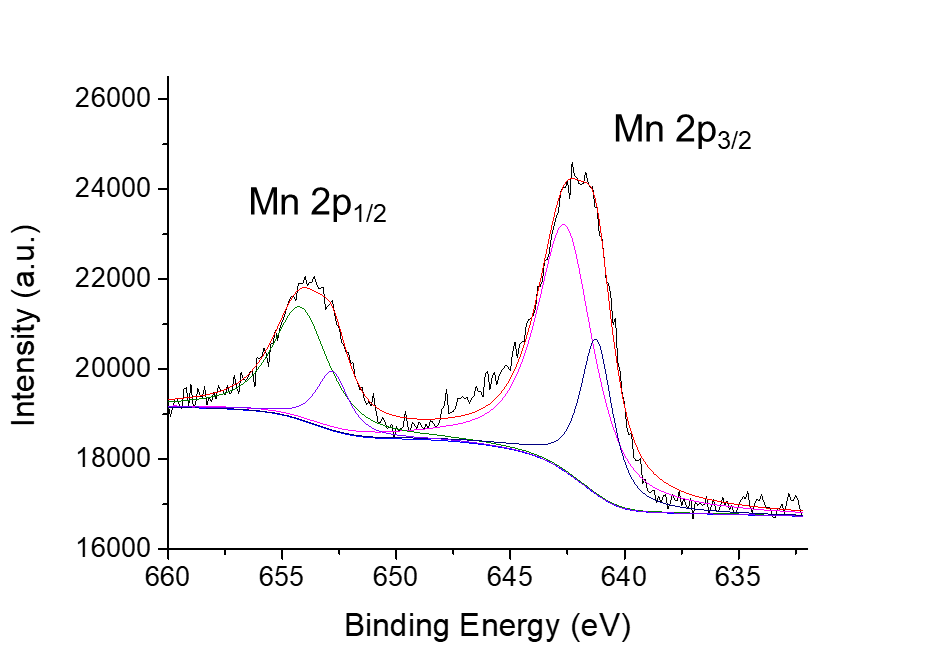


**Fig. S9.** X-ray Photoelectron Spectroscopy of PM-HMSN after US treatment.


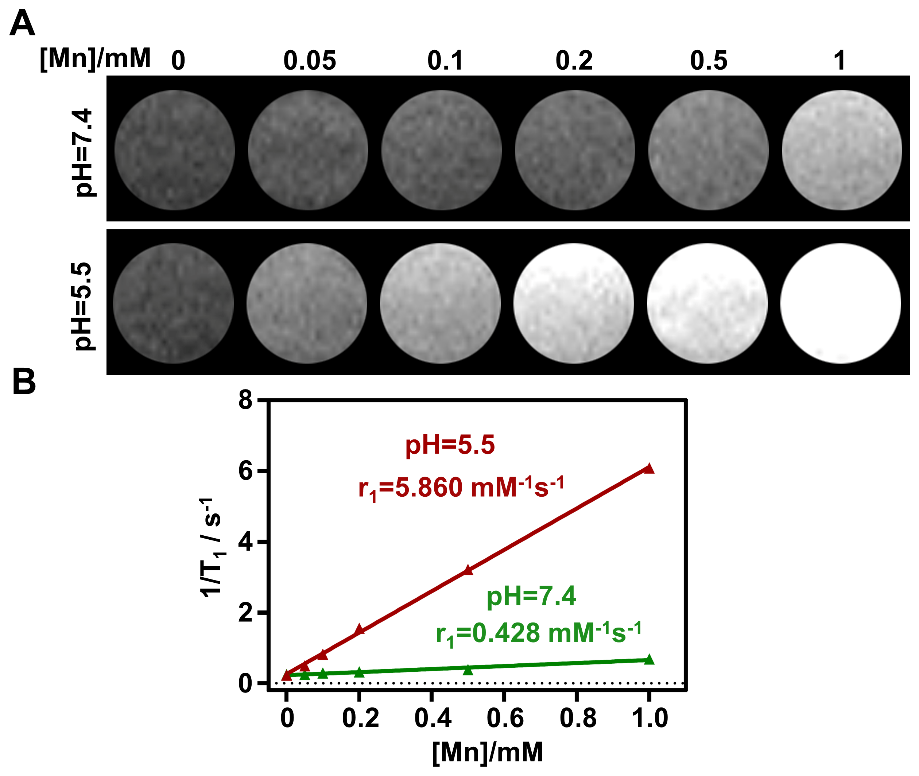


**Fig. S10.** *In vitro* T1-MRI of PM-HMSN/Arg. (A) T1-weighted MRI of PM-HMSN/Arg of different concentrations under different pH conditions. (B) T1-relaxation rate r_1_ vs Mn concentration for PM-HMS/Arg in different pH buffer solutions.

**
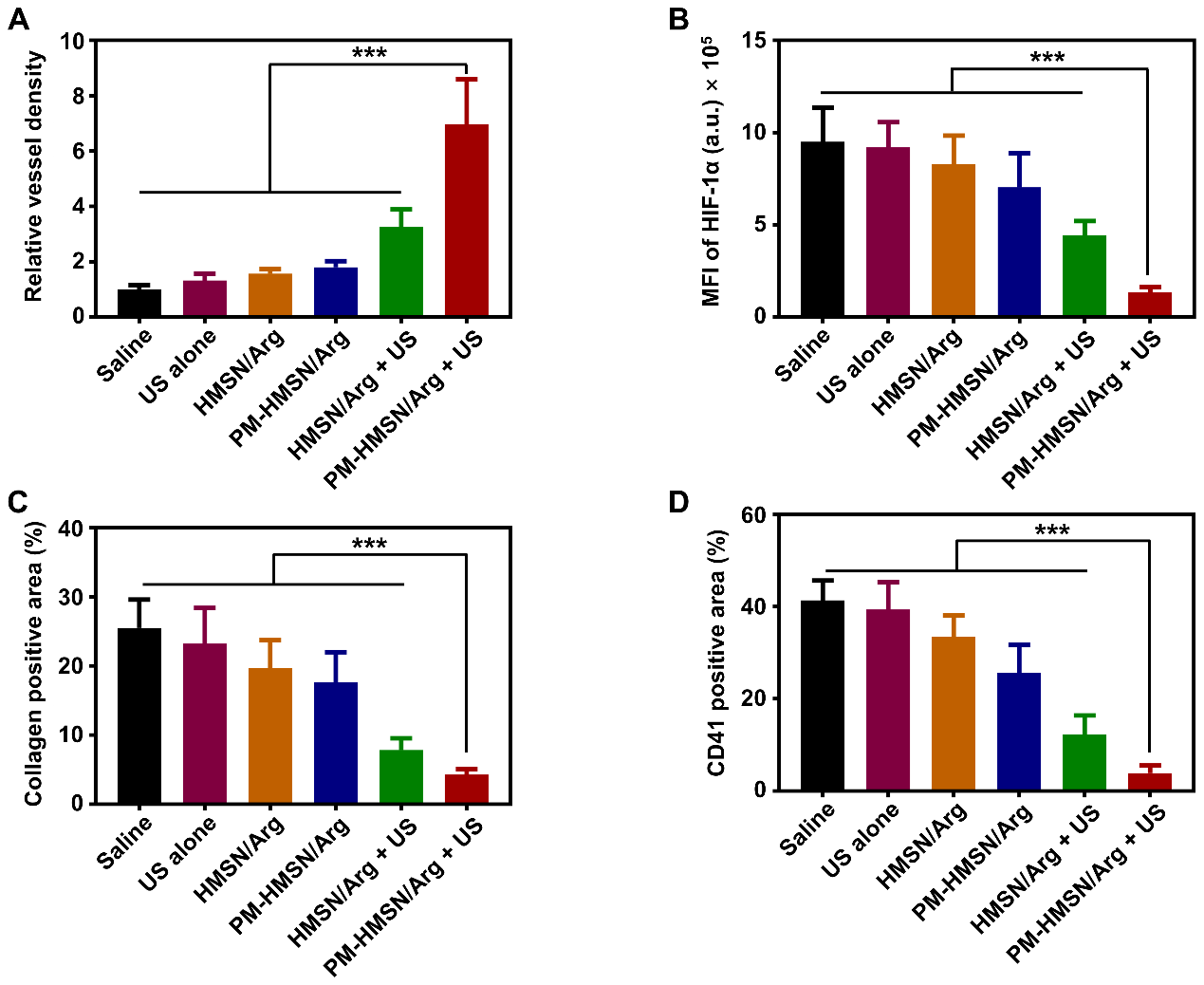
**

**Fig. S11.** Tumor microenvironment modulation effect of PM-HMSN/Arg through corresponding quantitative assays of (A) CD31, (B) HIF-1α, (C) type Ⅰ collagen, and (D) CD41 expression within tumor of the BxPC-3 subcutaneous xenograft bearing nude mice at the end of the treatment by ImageJ software. Data are presented as the mean ± SD (n = 6). ****p* < 0.001, among the marked groups using nonparametric two-tailed analysis of variance.


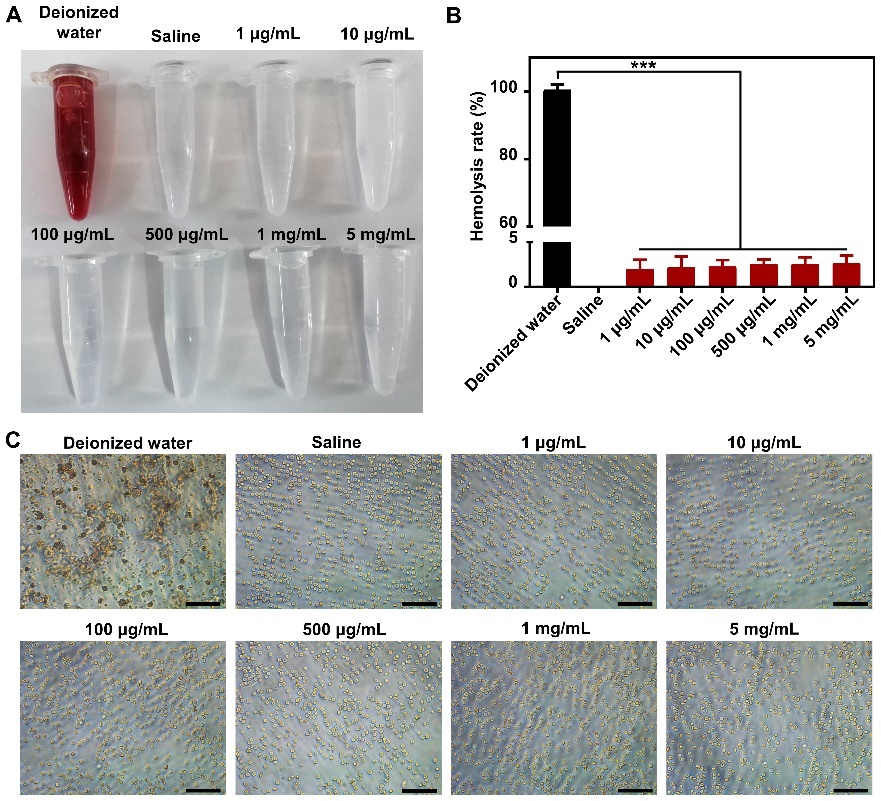


**Fig. S12.** The hemolysis assay of PM-HMSN/Arg. Red blood cells (RBCs) were incubated with the indicated formulations at different concentration at 37ºC for 2 h. Following centrifugation, (A) the supernatants were photographed and (B) the corresponding hemolysis rate was measured. Saline and deionized water were used for the negative and positive controls, respectively. Data are presented as mean ± SD (n = 3). ****p* < 0.001 among the marked groups using nonparametric two-tailed analysis of variance. (C) RBCs in the bottom post-centrifugation were also imaged. Scale bar: 200 μm.

**References**

[1] X. Jiang, T.L. Ward, Y.S. Cheng, J. Liu, C.J. Brinker, Aerosol fabrication of hollow mesoporous silica nanoparticles and encapsulation of L-methionine as a candidate drug cargo, Chemical communications (Cambridge, England), 46 (2010) 3019-3021.

[2] X. Pan, W. Wang, Z. Huang, S. Liu, J. Guo, F. Zhang, H. Yuan, X. Li, F. Liu, H. Liu, MOF-Derived Double-Layer Hollow Nanoparticles with Oxygen Generation Ability for Multimodal Imaging-Guided Sonodynamic Therapy, Angewandte Chemie, 59 (2020) 13557-13561.

[3] S. Li, Y. Li, H. Chen, Y. Yang, Y. Lin, T. Xie, N-Doped TiO(2) Coupled with Manganese-Substituted Phosphomolybdic Acid Composites As Efficient Photocatalysis-Fenton Catalysts for the Degradation of Rhodamine B, Langmuir : the ACS journal of surfaces and colloids, 38 (2022) 15817-15826.

[4] B. Li, Z. Gu, N. Kurniawan, W. Chen, Z.P. Xu, Manganese-Based Layered Double Hydroxide Nanoparticles as a T(1) -MRI Contrast Agent with Ultrasensitive pH Response and High Relaxivity, Advanced materials (Deerfield Beach, Fla.), 29 (2017).

[5] J.M. Argilés, J. Azcón-Bieto, The metabolic environment of cancer, Molecular and cellular biochemistry, 81 (1988) 3-17.

[6] X. Li, H. Zhou, Z. Niu, K. Zheng, D. Niu, W. Zhao, X. Liu, W. Si, C. Li, P. Wang, J. Cao, Y. Li, G. Wen, In Situ 3D-to-2D Transformation of Manganese-Based Layered Silicates for Tumor-Specific T(1)-Weighted Magnetic Resonance Imaging with High Signal-to-Noise and Excretability, ACS applied materials & interfaces, 12 (2020) 24644-24654.
